# Interaction of secondary ventricular tricuspid regurgitation with RV in HFREF: an invasive pressure-volume loop study

**DOI:** 10.1093/eschf/xvag134

**Published:** 2026-05-11

**Authors:** Alexander Schmeisser, Thomas Rauwolf, Thomas Groscheck, Tarek Bekfani, Katharina Fischbach, Blerim Luani, Ivan Tanev, Michael Hansen, Saskia Meißler, Paul Steendijk, Ruediger C Braun-Dullaeus

**Affiliations:** Division of Cardiology and Angiology, Department of Internal Medicine, Magdeburg University, Leipziger Str. 44, Magdeburg D-39120, Germany; Division of Cardiology and Angiology, Department of Internal Medicine, Magdeburg University, Leipziger Str. 44, Magdeburg D-39120, Germany; Division of Cardiology and Angiology, Department of Internal Medicine, Magdeburg University, Leipziger Str. 44, Magdeburg D-39120, Germany; Division of Cardiology and Angiology, Department of Internal Medicine, Magdeburg University, Leipziger Str. 44, Magdeburg D-39120, Germany; Department of Radiology, Magdeburg University, Magdeburg, Germany; Department of Cardiology, Klinikum Ingolstadt, Ingolstadt, Germany; Division of Cardiology and Angiology, Department of Internal Medicine, Magdeburg University, Leipziger Str. 44, Magdeburg D-39120, Germany; Division of Cardiology and Angiology, Department of Internal Medicine, Magdeburg University, Leipziger Str. 44, Magdeburg D-39120, Germany; Division of Cardiology and Angiology, Department of Internal Medicine, Magdeburg University, Leipziger Str. 44, Magdeburg D-39120, Germany; Department of Cardiology, Leiden University Medical Center, Leiden, The Netherlands; Division of Cardiology and Angiology, Department of Internal Medicine, Magdeburg University, Leipziger Str. 44, Magdeburg D-39120, Germany

**Keywords:** Tricuspid regurgitation, HFrEF, Right ventricular pressure–volume loop

## Abstract

**Introduction:**

Heart failure with reduced ejection fraction (HFrEF) accompanied by moderate or severe ventricular tricuspid-valve leaflet regurgitation (vTR2/3) is prognostically unfavourable; however, the underlying pathophysiology has not yet been sufficiently clarified. The hypothesis of a causative role of left ventricular (LV) dysfunction +/− secondary mitral regurgitation (sMR) on the extent and severity of secondary vTR was investigated.

**Methods:**

We integrated right ventricular (RV) pressure–volume loop and Swan-Ganz catheter data with RV/LV imaging findings in a retrospective analysis of 134 HFrEF patients.

**Results:**

Parameters independently associated with the presence of vTR2/3 were (i) presence of sMR (adjusted odds-ratio [aOR] = 1.67, *P* = .045), (ii) increased pulmonary vascular pulsatile RV loads (lower pulmonary artery [PA] compliance, aOR = 0.43, *P* = .021; area under the curve [AUC] = 0.82, cut-off <2.24 ml/mmHg, *P* < .001), mainly due to concomitant moderate/severe sMR (sMR2/3) (aOR = 4.56, *P* = .012), and (iii) progressive uncoupling of RV elastance/contractility (Ees) to an increasing total afterload (pulmonary elastance, Ea) (Ees/Ea ratio: aOR = 0.024, *P* = .005; AUC = 0.84, cut-off <0.6, *P* < .001). In addition, the RV-PA uncoupling was not only determined by the higher afterload in vTR2/3, but was also observed across the entire total afterload range (Ea tertile). This resulted in a larger and more dysfunctional RV in vTR2/3 compared with vTR0/1, independent of the afterload. RV-PA uncoupling and reduced PA compliance were independently associated with all-cause mortality.

**Conclusion:**

The vTR2/3 in context of HFrEF was independently associated with the presence of sMR, increased pulsatile loads, and a pronounced RV-PA uncoupling over almost the entire afterload range. Future studies will need to determine under which haemodynamic conditions a mechanical tricuspid regurgitation reduction in HFrEF patients is advisable.

## Graphical Abstract

**Graphical Abstract:**
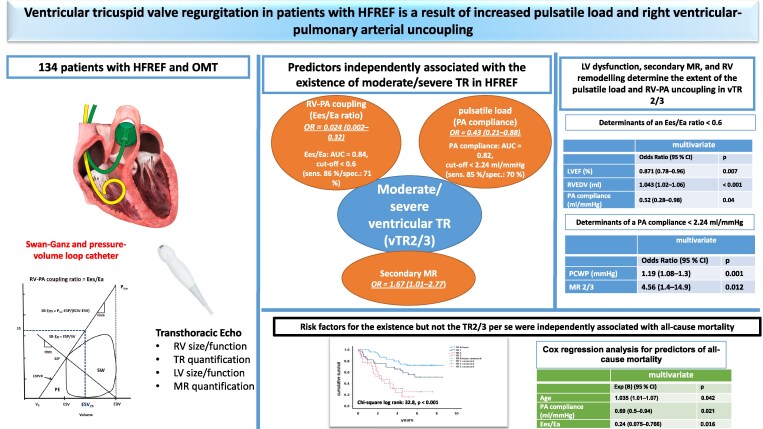

**Interaction of secondary ventricular tricuspid regurgitation with RV mechanics.**

## Introduction

Moderate or severe tricuspid regurgitation (TR) is associated with increased morbidity and mortality.^1–3^ The predominant mechanism of TR is secondary and can be classified as ventricular (in 60%–80% of patients, ventricular TR [vTR]) or atrial TR (aTR).^4,5^ The latter has prognostic and probably therapeutic implications, as vTR is associated with a significantly higher risk of death or hospitalization for heart failure than the atrial form.^6^ Ventricular TR in patients often occurs in the context of heart failure with reduced ejection fraction (HFrEF) and concomitant left-sided valvular diseases. In this context, vTR is mostly characterized by the dilatation and dysfunction of the right ventricle (RV), resulting in annular dilatation and, more importantly, apical displacement of the papillary muscles and tethering of the leaflets.^4^ The RV dilatation and dysfunction are most frequently due to remodelling in the context of pulmonary hypertension (PH), a fact that is best described pathophysiologically as an uncoupling of the intrinsic RV contractility (RV elastance, Ees) to afterload (pulmonary artery [PA] elastance, Ea).^7^ Nevertheless, increased pulmonary pressure does not appear to be the sole cause, as 34%–56% of patients with relevant PH have no or only mild TR.^8^ To the best of our knowledge, invasive pressure-volume (PV) loop data, as the gold standard of RV-PA coupling analysis, do not yet exist in the context of HFrEF-induced vTR. A comprehensive haemodynamic and imaging analysis using this technique is needed to better elucidate the relationship between HFREF +/−sMR and secondary vTR beyond elevated pulmonary pressures. The study examines the significant clinically unresolved inquiry whether vTR represents merely an epiphenomenon of PH or a more intricate haemodynamic cascade involving RV-PA uncoupling, increased pulmonary vascular pulsatile (PA compliance) and/or steady-state load (PVR), concurrent secondary mitral regurgitation (sMR), and progressive RV remodelling. The objective was to investigate the role of HFREF +/−sMR on RV mechanics and the extent and severity of secondary vTR. We integrated the invasive RV-PV loop technique and Swan-Ganz catheter data with RV/left ventricular (LV) imaging findings to analyse the impact of pulmonary-vascular loading conditions on RV contractility coupling and its association with RV remodelling and TR severity. Additionally, we analysed the correlation between these factors and patient prognosis.

## Methods

This *post hoc* analysis is based on 111 patients with HFrEF from the Magdeburg CRT (cardiac resynchronization therapy) Responder study (German Clinical Trial Register DRKS00011133; 2010–2016), who had baseline echocardiography, Swan-Ganz catheter, and RV-PV loop measurement. A magnetic resonance imaging (MRI) was carried out in a subgroup of patients (*n* = 50) without a pre-implanted device. Patients with symptoms of heart failure despite guideline-directed treatment, sinus rhythm, LV systolic dysfunction (EF ≤35%), and markers of cardiac dyssynchrony (QRS >130 ms) were included in this prospective monocentric exploratory study.^7^ After baseline examination, all patients received a CRT. The proportion of moderate and severe vTR was relatively low (23 from 111 patients) within this study, therefore, the study group (Group 1) was pooled for our current analysis with an additional group of 23 HFREF (LVEF ≤35%) patients (Group 2) from a local exploratory haemodynamic PV loop and Swan-Ganz catheter guided CRT-optimization trial (2020–2021), who were characterized by a haemodynamic and clinical non-response 6–12 months (LVEF ≤35%, NYHA III–IV) after cardiac resynchronization therapy. This clinical examination programme was cancelled due to a lack of success. Despite the long-time interval between the examinations, we pooled both patient groups because of very similar including (EF ≤35%, NYHA III–IV, and QRS > 130 ms, in Group 2 at baseline before CRT) and excluding criteria and examination procedures. The groups differed in various clinical, morphological (LV, RV), and haemodynamic parameters. Patients in Group 2 were slightly but significantly older, had a longer history of HFREF, a lower LVEF, and a larger LV (LVEDV). Secondary pulmonary vascular afterload was mildly higher than in Group 1 (PVR, PA compliance, systolic PA pressure, pulmonary capillary wedge pressure [PCWP]), and the proportion of patients with moderate/severe vTR (13 from 23 patients) and sMR was higher (*P* < .05).

To enable comparable fluid status, all examinations were carried out within 2–3 days after admission.

A torrential TR was an exclusion criterion in both studies. In addition, vTR patients with transthoracic/transesophageal echocardiographic (TTE/TOE) proof of leaflet impingement by pre-implanted leads were excluded from analysis. The clinical follow-up was performed every 6 months.

Both studies were approved by the Institutional Review Board. All patients had provided written informed consent.

### Echocardiography

Echocardiographic analysis was performed by two independent specialists using IntelliSpace Software (Philips, The Netherlands). The RV functional analysis, tricuspid annular plane systolic excursion (TAPSE), and fractional area change (FAC), including the systolic and end-diastolic area, quantifying the sMR, and the forward stroke volume (SV) were done according to the European recommendations.^9^ The qualitative and semi-quantitative quantification of vTR was determined according to the current state-of-the-art.^10^

### Cardiac magnetic resonance (MRI)

A subgroup of patients (*n* = 50) without a pre-implanted device underwent MRI using a 1.5-T CMR scanner (Philips, The Netherlands) at baseline. Briefly, steady-state free precession cine images were acquired in multiple short- and long-axis views (ECG triggering, breath-hold technique). The analysis of RV was performed by determining the end-diastolic and -systolic frames on short-axis cine views (Circle cvi42, Canada).

### Left and right heart catheterization, RV-PV loop catheter

The left and right heart catheter measurements were determined as described previously.^11^ The mean of the indirect Fick method and the echo-derived velocity time integral method in the aortic outflow tract were used for forward cardiac output.

Pressure-volume loop measurement: A 7F RV-PV catheter (CD Leycom, The Netherlands) was positioned via the internal jugular vein for PV loop analysis. The PV loop was obtained (Inca©, CD Leycom, The Netherlands) and the data were analysed using Circlab (Leiden University, The Netherlands).^7^

### Statistics

All data and statistics are reported as median and interquartile range [25%–75%]. Categorical data were summarized by percentages. Baseline characteristics were compared using the Mann–Whitney *U* test. Binary multivariate regression analysis was used to identify parameters that are associated with the existence of vTR2/3. We tested potential predictors that could favour the presence of vTR2/3 through local mechanical interactions (implanted leads), or through influences on RV remodelling, such as LV–RV interaction, LVEF, RV mechanics (RV size), and RV afterload (sMR, LA size, and parameters of pulmonary vascular afterload, such as PA compliance, PVR, Ea, and PCWP). The ROC analysis was used to derive a precise cut-off of Ees/Ea and PA compliance (Youden index) that would best discriminate the existence of vTR2/3 compared to vTR0/1. Similarly to the method used to detect vTR 2/3, we searched for haemodynamic and morphological predictors of PA compliance and an Ees/Ea ratio below the cut-offs calculated by the ROC analysis. We always test for multicollinearity before running the logistic regression model. Multicollinearity among independent variables was assessed using the variance inflation factor and tolerance values by linear regression. All predictor variables had variance inflation factor values below 5 and tolerance values above 0.2. We used the correlation matrix in the final binary regression model to exclude independent predictors with an *r* > 0.8.

The Kaplan–Meier method was used to compare the different strata of vTR compared with the log-rank test. Cox regression analysis was used for the independent mortality predictors. Variables perceived as clinically important and those with a <0.1 in univariate analysis were included in the multivariate model. Differences with a *P* < .05 were considered statistically significant. Due to the pooling of two patient groups with identical inclusion criteria but some differing baseline characteristics, we performed a detailed cohort-specific sensitivity analysis (Supplementary Sensitivity Analysis Tables and analysis figures).

## Results

**Figure 1:**
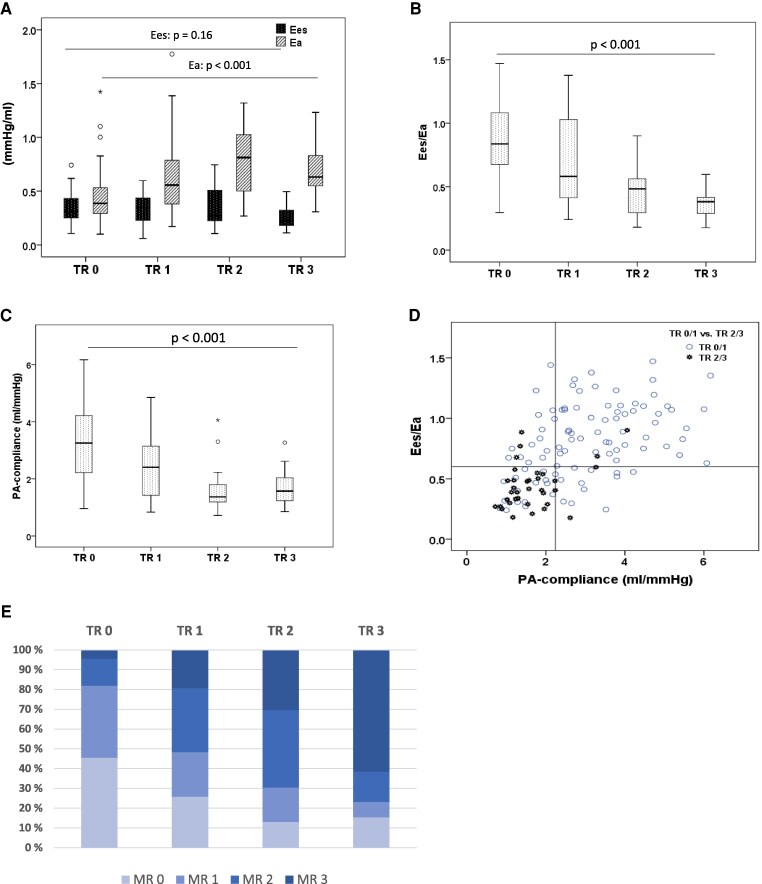
Box plot analyses show a steady rise in total PA afterload Ea, no adaptive increase of Ees (*A*), and a lowering of Ees/Ea (*B*) and PA compliance over vTR strata (*C*). (*D*): Scatter plot of the relationship of Ees/Ea vs. PA compliance in all patients. The horizontal line marks the cut-off of the Ees/Ea ratio 0.6. The vertical line is the cut-off of PA compliance at 2.24 ml/mmHg. Hollow circles denote patients with vTR0/1. The jagged, darkly highlighted circles, of which 80% are located in the stratum Ees/Ea < 0.6 and PA compliance <2.24 mmHg/ml, denote vTR2/3 patients. (*E*) Concomitance of MR 0–3 according to the severity of vTR. The occurrence of sMR 2/3 rise steadily significantly with the vTR strata. (TR: tricuspid regurgitation; Ees: right ventricular end-systolic elastance; Ea: pulmonary artery elastance; PA: pulmonary artery; RV: right ventricular; sMR: secondary mitral regurgitation)

A total of 36 out of 134 HFrEF patients presented with vTR2 (*n* = 23, 17.4%) or vTR3 (*n* = 13, 9.7%). The latter group included two patients with massive TR. A vTR1 was observed in 32 (23.9%) patients (*Table 1*). The clinical characteristics, haemodynamic measures, and echocardiographic imaging data are presented, according to the severity of the vTR, in Supplementary Tables S1 and S2, *Figure 1*, and Supplementary Figure S2.

**Table 1:** Baseline characteristics

Baseline characteristics	Measure (N = 134)
**Age (years)**	68 (60–74)
**Men (%)**	86.2
**NYHA III (%)**	86.4
**ICM (%)**	51.4
**Permanent/persistent atrial fibrillation (%)**	7
**Diuretics (%)**	87
**ACE Inhibitors/ARBs/ARNI (%)**	95
**B-Blockers (%)**	97
**Aldosteron-I. (%)**	52
***LV Parameter***	
**LV-EF (ml)**	30 (25–35)
**LVEDV (ml)**	215 (188–274)
**LA volume (ml)**	91 (67–120)
**MR 2/3, N (%)**	54 (41)
***Swan-Ganz catheter***	
**PA mean (mmHg)**	28 (33–39)
**PCWP mean (mmHg)**	19 (13–27)
**PVR (dyn.)**	193 (132–265)
**PA compliance (ml/mmHg)**	2.39 (1.57–3.74)
***TR (echo)***	
**TR 0/trace N (%)**	66 (49.3)
**TR mild (I) N (%)**	32 (23.9)
**TR moderate (II) N (%)**	23 (17.4)
**TR severe/massive (III) N (%)**	11/2 (9.7)

Median (25/75th percentile)

ACE-I, angiotensin converting enzyme inhibitor; AT, angiotension receptor; MRA, mineralocorticoid receptor antagonist; ICM, ischemic cardiomyopathy; PA_mean_, mean pulmonary arterial pressure; PH, pulmonary hypertension; LVEDV, left ventricular end-diastolic volume; LVEF, LV ejection fraction; LVEDP, left ventricular end-diastolic pressure; MR, mitral regurgitation; LA, left atrium; PVR, pulmonary vascular resistance; PCWP, pulmonary capillary wedge pressure; TR, tricuspid regurgitation

### Haemodynamic and morphologic parameters independently associated with the presence of moderate/severe vTR (vTR2/3)

We found that the existence of concomitant sMR, a lower PA compliance as a measure of the increased RV pulsatile load, and a pronounced haemodynamic RV-PA uncoupling (lowered PV loop derived Ees/Ea ratio) remained independently associated with the presence of vTR2/3 (*Table 2*) in multivariate binary logistic regression analyses, including parameters of LV performance, sMR severity, pulmonary–vascular haemodynamics, invasive and non-invasive RV-PA performance, and coupling analysis. An Ees/Ea ratio cut-off of <0.6 (AUC 0.84, *P* < .001) with a sensitivity and specificity of 86 and 71%, respectively, and a PA compliance of <2.24 ml/mmHg (AUC 0.82, *P* < .001) with a sensitivity and specificity of 85% and 70%, respectively, discriminated between vTR0/1 and vTR2/3 in the receiver operating characteristic analysis (ROC) (Supplementary Sensitivity Analysis Figures). In fact, 80% of our patients with vTR2/3 were characterized by a combination of an Ees/Ea ratio <0.6 and a PA compliance <2.24 ml/mmHg (*Figure 1E*). Interestingly, a PA compliance <2.24 ml/mmHg was independently linked to the presence of moderate or severe sMR (sMR2/3) and a consecutively elevated LA pressure (PCWP) in the logistic regression analysis (*Table 3*; in addition, pulmonary vascular haemodynamics according to the severity of sMR is shown in Supplementary Figure S3).

**Table 2:** Lowered PA compliance and RV Ees/Ea ratios, and the occurrence of secondary MR are independently associated with vTR2/3 in patients with HFREF in the multivariate binary logistic regression analysis

	Univariate		Multivariate	
	Odds Ratio (95% CI)	*P*	Odds Ratio (95% CI)	*P*
PM/AICD/CRT (%)	3.1 (1.24–7.77)	.015		
PCWP mean (mmhg)	1.11 (1.06–1.17)	<.001		
PVR (dyn)	1.006 (1.003–1.009)	<.001		
PA compliance (ml/mmHg)	0.26 (0.147–0.47)	<.001	0.43 (0.21–0.88)	.021
LV-EF (%)	0.9 (0.86–0.97)	.002		
LA volume diastolic (ml)	1.02 (1.01–1.02)	.001		
Ees/Ea	0.004 (0.001–0.035)	<.001	0.024 (0.002–0.32)	.005
sMR (0–3)	6.4 (2.7–15)	<.001	1.67 (1.01–2.77)	.045
RVEDV (ml, PV loop)	1.027 (1.015–1.039)	<.001		

TR, tricuspid regurgitation; ICM, ischemic cardiomyopathy; PM, pacemaker; AICD, automatic implantable cardioverter defibrillator; CRT, cardiac resynchronization therapy; PA, pulmonary artery pressure; PCWP, pulmonary capillary wedge pressure; PVR, pulmonary vascular resistance; LV-EF, left ventricular ejection fraction; LVEDP, left ventricular end-diastolic pressure; LA, left atrial; Ea, PA elastance; Ees, end-systolic elastance of the right ventricle; FAC, right ventricular fractional area change; TAPSE, tricuspid annular plane systolic excursion; PASP, systolic pulmonary arterial pressure; MR, mitral regurgitation; RVEDV, right ventricular end-diastolic volume; PV, pressure volume

**Table 3:** A higher PCWP and the occurrence of an sMR2/3 are independently associated with a PA compliance <0.6 mmHg/ml in the multivariate binary logistic regression analysis

	Univariate		Multivariate	
	Odds Ratio (95% CI)	*P*	Odds Ratio (95% CI)	*P*
LVEDP (mmHg)	1.13 (1.06–1.19)	<.001		
PCWP (mmHg)	1.24 (1.15–1.33)	<.001	1.19 (1.08–1.3)	.001
LA size (ml)	1.02 (1.009–1.032)	<.001		
LVEF (%)	0.93 (0.88–0.98)	.008		
Age (years)	1.06 (1.01–1.1)	.01		
sMR 2/3	6.6 (3–14.4)	<.001	4.56 (1.4–14.9)	.012

LVEDP, left ventricular end-diastolic pressure; PCWP, pulmonary capillary wedge pressure; LA, left atrial; LVEF, left ventricular ejection fraction; sMR, secondary mitral regurgitation

### Determinants and consequences of the vTR2/3-associated RV-PA uncoupling

**Figure 2:**
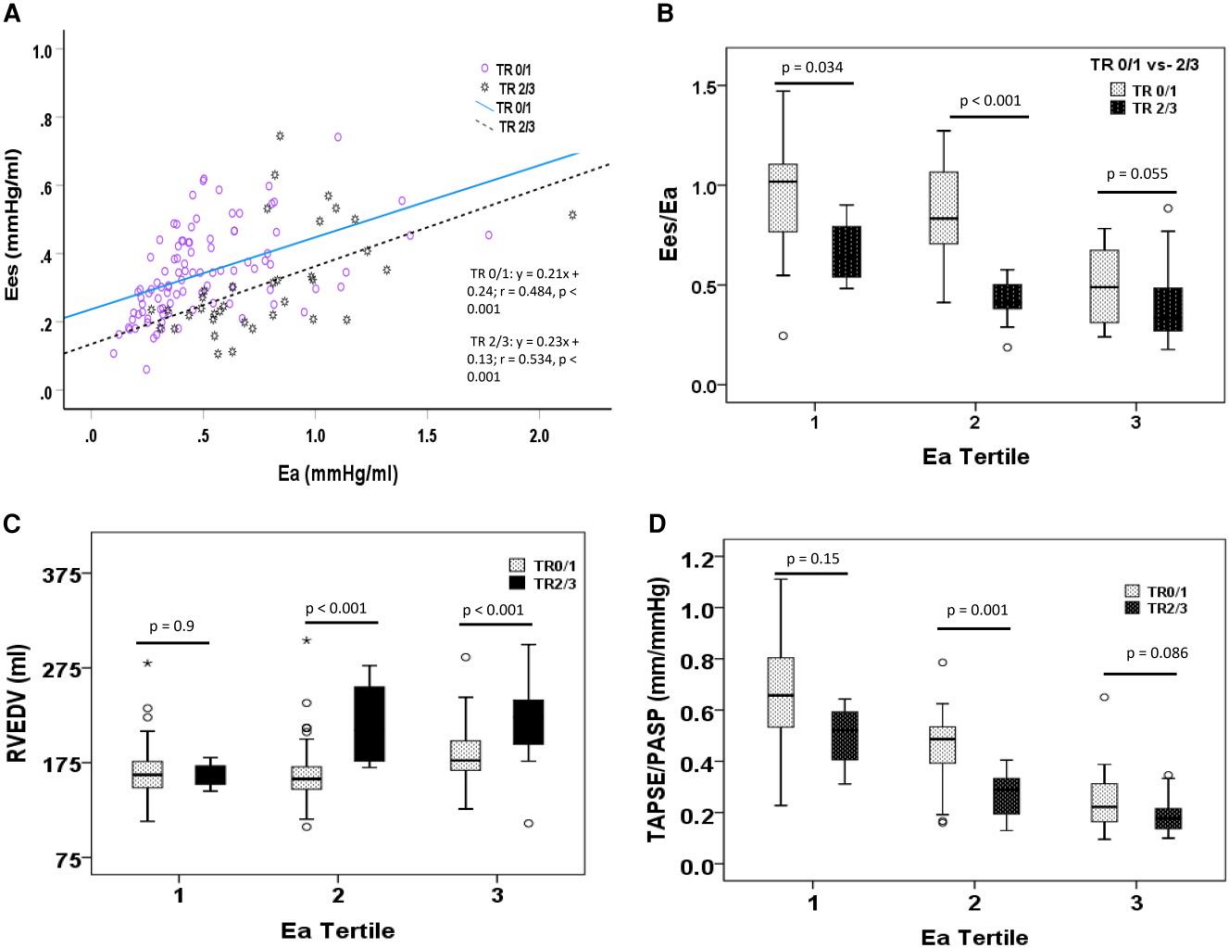
Non-adaptive RV contractility response (Ees) to the entire afterload range (Ea), and its relating parameter of RV remodeling and function according to the TR severity (vTR0/1 vs. vTR2/3) and comparable Ea tertile. (*A*). Scatterplot analyses of the relationship between intrinsic RV contractility and RV total afterload Ea. Hollow circles denote patients with vTR0/1. The jagged, darkly highlighted circles denote patients with vTR2/3. The straight line represents the regression line of vTR0/1. The dashed line is the regression line of vTR2/3 patients. vTR2/3 was associated with a lower capacity of RV contractility (Ees) adaptation across almost all total afterload ranges in comparison to vTR0/1, indicated by a downshift of the regression lines in comparison to TR0/1. (*B–D*) At comparably similar total afterload conditions (Ea tertile 1–3), vTR2/3 patients were almost always characterized by a lower RV-PA coupling ratio Ees/Ea and, consequently, a larger (RVEDV) and more dysfunctional, non-invasively measured, uncoupled RV (TAPSE/PASP). (Ea Tertile: Ea-1: Ea ≤0.38 mmHg/ml, Ea-2: >0.38 but <0.63 mmHg/ml, Ea-3: Ea ≥0.63 mmHg/ml). (TR: tricuspid regurgitation; Ees: right ventricular end-systolic elastance; Ea: pulmonary artery elastance; ME: mechanical efficiency: quotient stroke work/pressure volume area; FAC: fractional area change; RVEDV: right ventricular end-diastolic volume)

The progression from vTR0 to vTR3 was significantly associated with increasing pulsatile (declining PA compliance) and total (Ea) RV afterloads (*Figure 1*). The ability of the RV to adapt its intrinsic contractility (Ees) to an increasing total afterload (Ees/Ea ratio) declined from vTR0 to vTR3 (*Figure 1*, Supplementary Tables S1 and S2). This was accompanied by a progression of RV remodelling and dysfunction, lower mechanical RV efficiencies (ME), higher stroke works, and higher PV loop areas (PVA) (Supplementary Table S2, Supplementary Figure S2). The pronounced RV-PA uncoupling in vTR2/3 was not exclusively a function of increased afterloads in these patients. We observed that vTR2/3 was associated with a lower capacity of RV contractility (Ees) adaptation across almost all total and pulsatile afterload ranges in comparison to vTR0/1, indicated by a downshift of the regression lines in comparison to TR0/1 (regression analysis Ees-Ea: *Figure 2A*, Ees-PA compliance: Supplementary Figure S5). In addition, at comparable total afterload tertiles (Ea tertile 1–3), vTR2/3 patients were almost always characterized by a lower RV-PA coupling ratio Ees/Ea, and, consequently, a larger right ventricular end-diastolic volume (RVEDV) and more dysfunctional RV (TAPSE/pulmonary artery systolic pressure [PASP]) with a lower mechanical RV efficiency and higher PVA than vTR0/1 patients (*Figure 2*, Supplementary Figure S3). A significantly reduced systolic LV–RV interaction, among other factors, may be jointly responsible for the RV-PA uncoupling in especially vTR2/3 patients. We found in the regression analysis that a lower LVEF was an independent predictor of the vTR2/3-associated RV-PA uncoupling (Ees/Ea < 0.6) (*Table 4*). In fact, vTR2/3 showed a lower LVEF than vTR0/1 patients (*P* = .001).

**Table 4:** A lower LVEF, larger RV, and lower PA compliance are independently associated with a pronounced RV-PA uncoupling (Ees/Ea < 0.6) in the multivariate binary logistic regression analysis

	Univariate		Multivariate	
	Odds Ratio (95% CI)	*P*	Odds Ratio (95% CI)	*P*
LVEDP (mmHg)	1.08 (1.02–1.1)	.006		
PCWP (mmHg)	1.09 (1.06–1.16)	<.001		
TPG (mmHg)	1.1 (1.03–1.2)	.006		
LVEF (%)	0.88 (0.83–0.93)	<.001	0.871 (0.78–0.96)	.007
RVEDV (ml)	1.044 (1.03–1.06)	<.001	1.043 (1.02–1.06)	<.001
Age (years)	1.039 (0.99–1.08)	.056		
PA compliance (ml/mmHg)	0.28 (0.18–0.45)	<.001	0.52 (0.28–0.98)	.04
PVR (dyn.)	1.008 (1.004–1.01)	<.001		

LVEF, left ventricular ejection fraction; LVEDP, left ventricular end-diastolic pressure; PCWP, pulmonary capillary wedge pressure; TPG, transpulmonary gradient; Ea, PA elastance; RVEDV, right ventricular end-diastolic volume; PVR, pulmonary resistance

### Survival analysis

**Figure 3:**
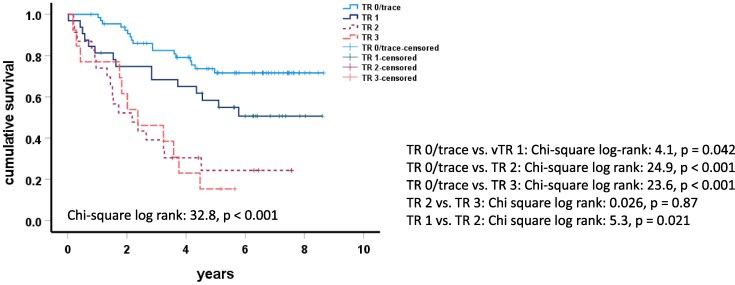
Kaplan-Meier plots of outcome: patients were stratified according to the severity of TR, which revealed significant differences in the overall survival over all strata (Chi-square log rank: 32.8, *P* < .001)

A total of 75 (55.97%) of the 134 patients died during the median follow-up of 4.39 years (1.96–6.44). The Kaplan–Meier analysis showed that the severity of vTR further discriminated all-cause mortality (chi-square log rank: 32.8, *P* < .001) (*Figure 3*). The all-cause mortality in HFREF patients with vTR2 or 3 was not statistically different from each other, but increased dramatically in comparison to vTR0 or 1.

A multivariate Cox regression analysis demonstrated that only age, the RV-PA coupling ratio Ees/Ea, and PA compliance were independently predictive of a long-term prognosis (Model 1: Supplementary Table S3). The presence of vTR2/3 remained independently associated with all-cause mortality only after exclusion of all haemodynamic coupling parameters (Model 2: Supplementary Table S4).

## Discussion

To the best of our knowledge, this is the first study using the gold standard of RV functional analysis, the invasive RV-PV loop technique, in combination with a Swan-Ganz catheter and non-invasive imaging data to examine the impact of RV-PA coupling and its confounders on the presence of vTR2/3 in HFREF patients. The occurrence of secondary vTR2/3 was independently associated with the existence of concomitant sMR, with secondary increased pulmonary vascular pulsatile RV loads (lower PA compliance), which, in turn, were mainly due to concomitant moderate/severe sMR and a consecutive progressive uncoupling of RV intrinsic contractility (Ees) to total afterload (pulmonary elastance, Ea).

Recent data from RV-PV loop studies have shown that the RV size and function are very closely regulated by the ratio of end-systolic RV elastance (Ees) to PA elastance (Ea) (RV-PA coupling) in precapillary idiopathic pulmonary arterial hypertension patients and post-capillary PH due to HFrEF.^12–14^ In concordance with the significantly reduced Ees/Ea ratio, vTR2/3 patients in our cohort were characterized by a larger and more dysfunctional RV with higher PVAs (as a surrogate for increased RV oxygen consumption) and lower mechanical efficiency than patients with vTR0 or 1. As has been shown recently, the height of total afterloads in patients with HFREF and secondary PH is associated with RV dyssynchrony,^15^ and one of the main regulators of the efficiency of RV-PA coupling (Ees/Ea ratio).^7,16^ The pronounced RV-PA uncoupling in our vTR2/3 cohort was not solely attributable to their higher afterloads per se when compared with vTR0/1 patients. We observed that vTR2/3 patients at comparable total afterload tertiles (Ea tertile 1–3) were almost always characterized by lower RV-PA coupling ratios Ees/Ea and, consequently, larger (RVEDV) and more dysfunctional RVs (ME, PVA, FAC, TAPSE/PASP) than TR0/1 patients. Among other factors, such as intrinsic RV muscle damage due to the involvement in the LV disease process, significantly reduced systolic LV–RV interaction may be jointly responsible for the RV-PA uncoupling.^7,17^ The regression analysis showed that a lower LVEF was an independent predictor of the vTR2/3-associated pronounced RV-PA uncoupling (Ees/Ea < 0.6). Indeed, vTR2/3 patients had a lower LVEF than vTR0/1 patients.

In addition to an advanced RV-PA uncoupling, a higher pulsatile RV load per se (lower PA compliance) and the presence of a significant sMR were significantly and independently associated with the presence of vTR2/3 in our HFREF cohort. The association of a significant sMR with secondary vTR in patients with LV dysfunction has been shown previously.^18^ As this and other studies have been based exclusively on non-invasive echocardiographic, the haemodynamic connection between the existence of sMR and vTR development of has not been clarified. Moderate or severe sMR (sMR2/3) accompanied vTR2/3 in 72.2% of cases in our pooled HFrEF cohort. We found in the logistic regression analysis that sMR2/3 and consecutively increased left atrial pressures (PCWP) were independently associated with increased pulsatile RV loads (reduced PA compliance), which, in turn, mainly predicted the occurrence of vTR2/3. In addition, it has recently been shown that higher LA pressures may change the hyperbolic PA compliance-pulmonary vascular resistance relationship to an enhanced pulsatile relative to the resistive load.^19^ The amount of pulsatile load independently affected the RV-PA coupling capacity, which may lead to further RV dilatation and dysfunction out of proportion to the total afterloads in patients with HFREF and secondary PH.^7^ Based on these data, we suspect that a reduced PA compliance, as an expression of a higher pulsatile RV afterload, may be an important haemodynamic link between sMR2/3 and the secondary occurrence of vTR2/3 in HFREF. In addition, an increased pulsatile load may further compromise the RV-PA coupling capacity and the associated RV remodelling process in the context of HFrEF complicated by vTR2/3.

Moderate or severe secondary vTR is associated with increased morbidity and mortality.^1,2^ Our Kaplan–Meier data confirm a dramatically reduced survival in vTR2/3 patients. The results regarding an independent association between vTR and mortality in HFrEF patients are more contradictory. Functional secondary TR in HFrEF in a large cohort was associated with considerably worse survival, independent of the baseline parameters.^18^ Recent data have emphasized that the prognostic impact of TR depends on the severity of systolic heart failure and is linked to mortality only in patients with mild heart failure, but provides no additive value in advanced disease.^20^ All these studies were based only on clinical and echocardiographic data. Using a Cox regression model including both haemodynamic Swan-Ganz and RV-PV loop, we found that only the haemodynamic causes of vTR2/3, such as the RV-PA coupling ratio Ees/Ea and the PA compliance, but not vTR2/3 per se, were independently associated with all-cause mortality in more advanced HFrEF.

What might be the implications of a targeted therapy of moderate or severe TR in patients with HFrEF based on the specific pathologic findings that we have found in this analysis?

The trigger factors in our analysis determine the subsequent prognosis in HFrEF and not the moderate or severe vTR itself. The former are mostly secondary consequences of LV dysfunction and concomitantly significant sMR. Accordingly, the therapeutic focus should initially be directed towards the LV performance and secondary MR. This includes an optimized drug and device therapy of HFrEF patients.^21,22^ An additional interventional reduction of functional MR by transcatheter edge-to-edge repair may be associated with a significant decrease of RV afterload (PASP), resulting in an improvement of non-invasively determined RV-PA coupling surrogate parameter TAPSE/PASP in 66% of cases.^23^ The question of whether substantial TR, in the context of HFrEF that persists after the optimization of LV and valvular conditions, should be mechanically reduced to improve the clinical outcome and prognosis remains uncertain. The majority of studies that could show a clinical improvement after successful TTVR mostly included patients with aTR, usually associated with normal LVEF and preserved RV function.^24,25^ The retrospective Trivalve registry (mean LVEF 50%) found that a pronounced RV-PA uncoupling at baseline, measured by TAPSE/PASP ratio of <0.406 was associated with worse survival after TTVR, also mostly in patients with aTR.^26^ A baseline TAPSE/PASP >0.44 was suggested in an exploratory meta-analysis.^27^ The median TAPSE/PASP in moderate and severe vTR was extensively lower, with 0.21 and 0.17 mm/mmHg, respectively, in our study. Only 3 out of 36 patients (8%) were characterized by a prognostic favourable TAPSE/PASP >0.406 mm/mmHg. An improvement of symptoms and quality of life could be observed with a more or less complete TR elimination using the Evoque valve in the TRISCENT II Study with a baseline TAPSE/PASP of 0.42, but this was associated with a decline of RV function (FAC 39.5–30.2%, TAPSE 16.2–12 mm) after 1 year of follow-up.^25^ In addition, within the Evoque valve in a real-world setting, including 29% vTR and 46% aTR, a baseline moderate or severe RV dysfunction was a strong predictor of adverse clinical outcomes (odds ratio [OR] 3.6, *P* = .008).^28^

A subgroup analysis of patients with more vTR in the controlled TRILUMINATE (14%) and the bRIGHT study (24% of patients included) has not yet been published. In addition, LVEF ≤35% was an exclusion criterion in TRI-FR,^29^ and an LVEF <25% in TRISCENT II.^25^ A comprehensive analysis and optimization of LV function, the severity of sMR also under exercise conditions, the pulmonary vascular haemeodynamics, the RV size and function, and the TR severity, including the patho-anatomy of the tricuspid valve (TV) (Hahn classification,^30^) are required in isolated vTR cases where a mechanical TR intervention is under consideration. The patho-anatomy of the TV is crucial for selecting the appropriate interventional treatment strategy, as achieving an optimal result (TR ≤ 1) must be the therapeutic goal.^9^ Only severe RV and LV dysfunction, precapillary PH, and/or severe PH (mean PAP >35 mmHg, PVR >5 Wood units) are considered contraindications for surgical or interventional TV therapy in the 2025 guidelines for valvular heart disease.^9^ Several analyses have suggested more conservative cut-off values, particularly for RV function and RV afterload. The frequently used TAPSE/PASP ratio as a surrogate parameter for RV–PA coupling has only limited value, as its correlation with invasively measured Ees/Ea ratios is, at best, moderate.^11,31^ An optimal threshold for systolic PA pressure appears to be <46 mmHg.^32^ Optimal prognostic thresholds identified in the EuroTR registry for death and heart failure hospitalization at two years, or for early clinical deterioration with signs of RV failure were: mean PA pressure ≥32 mmHg, PCWP ≥20 mmHg, and pulmonary vascular resistance ≥5 Wood units.^33^

Impaired RV function prior to TR intervention also has an impact on long-term survival. Identified cut-offs include an RV ejection fraction ≥45%, TAPSE ≥17 mm, and RV-FAC ≥35%.^34^ What level of LV function is required in such cases has not been systematically investigated to date. LV function is an important determinant of RV contractility, mostly due to intrinsic systolic ventricular interaction, based on our own experience, and LVEF <30% appears to be unsuitable.

Our study has several limitations. This is a *post hoc* analysis of a pooled patient cohort including a single-centre prospective observational study and a clinical examination programme. No patients with torrential and only two patients with massive TR were included. In addition, our study included 86 (64%) patients with pre-implanted RV leads. Relevant leaflet impingements by leads have been excluded by a comprehensive echocardiography prior to study inclusion. The calibration of the SV and RVEDV in PV loops outside the MRI subgroup (*n* = 50) was based on the combined indirect Fick method and echo velocity time integral measurement. The single-beat estimation of Ees/Ea, as opposed to multi-beat validation, seems to be a weakness of the study. It was shown in different validation studies that a reasonable agreement between the single-beat and the multi-beat RV Ees/Ea determination was established, at least when the ratio is <1, in different pre-capillary PH cohorts.^31,35,36^

Due to the *post hoc* design and the combination of two patient groups (Group 1, *n* = Group 2, *n* = 23), which were characterized by identical inclusion and exclusion criteria and baseline assessments, but were different in some baseline characteristics, we conducted a cohort-specific sensitivity analysis to increase confidence in the relevance of study results based on the pooled data. The different cohort-specific analyses are listed in the Supplementary Sensitivity Analysis Tables S1–S4, Supplementary Tables S1–S4 (for each group), and Sensitivity analysis figures. In summary, the main results of the pooled data could essentially be reproduced by the isolated analysis of Group 1. This applies both to the predictors for the presence of a vTR2/3 and the haemodynamic parameters underlying it, namely, low compliance and RV-PA un-coupling. The all-cause mortality is also generally associated with almost the same invasive and non-invasive parameters as in the pooled analysis.

The interpretation of the results of Group 2 is somewhat more complex, because this group is five times smaller; consequently, the number of patients within the vTR strata was very small. The analyses showed at least a partially strong trend towards similar results. The lowering of Ees/Ea and PA compliance and the increase of sMR2/3 could also be observed in Group 2, but were not statistically significant. RV-PA uncoupling (low Ees/Ea ratio) remains a strong predictor for the existence of vTR2/3. The lower, but statistically not significant, PA compliance in vTR2/3 patients was also associated with the existence of secondary MR, and the RV-PA uncoupling (low Ees/Ea) showed a significant association with a higher afterload (PCWP, PA compliance) in univariate analyses. The group and the number of patients within the vTR strata appeared too small for a meaningful Cox regression analysis.

In conclusion, combining the gold standard of RV functional analysis, the invasive RV-PV loop technique, with Swan-Ganz catheter and non-invasive imaging data, we found that the occurrence of secondary vTR2/3 in patients with HFREF is not only an epiphenomenon of secondary PH. In our analysis, vTR2/3 was independently associated with the existence of a concomitant sMR, consecutively increased pulmonary vascular pulsatile RV loads (lower PA compliance), which, in turn, were due mainly to concomitant moderate/severe sMR and a progressive uncoupling of RV intrinsic contractility (Ees) within the entire afterload range (pulmonary elastance, Ea). The significantly reduced LVEF in patients with vTR2/3 may further exacerbate RV–PA uncoupling due to inadequate LV–RV interaction.

Thus, the haemodynamic risk factors associated with the existence of vTR2/3 in HFrEF patients are determined, to a considerable extent, by the severity of LV dysfunction and concomitant mitral regurgitation. Consequently, the therapeutic focus in the context of vTR must primarily place emphasis on the improvement of LV and valvular function before considering the targeted mechanical reduction of TR. Future studies will need to determine whether and under which pulmonary vascular, LV, and RV conditions a mechanical TR reduction is advisable.

## Supplementary Material

xvag134_Supplementary_Data

## Declarations

### Data Availability

No data were generated or analysed for this manuscript.

### Pre-registered Clinical Trial Number

DRKS—German Clinical Trials Register (DRKS00011133)
