# Supplementary material for: Interaction of secondary ventricular tricuspid regurgitation with RV in HFREF: an invasive pressure-volume loop study
Source: ESC Heart Fail. 2026 May 11;13(3):xvag134. doi: 10.1093/eschf/xvag134 (PMC13220961; doi:10.1093/eschf/xvag134)
Supplement: xvag134_Supplementary_Data [file xvag134_supplementary_data.zip › 20_supplement figures.pptx]

## Slide 1
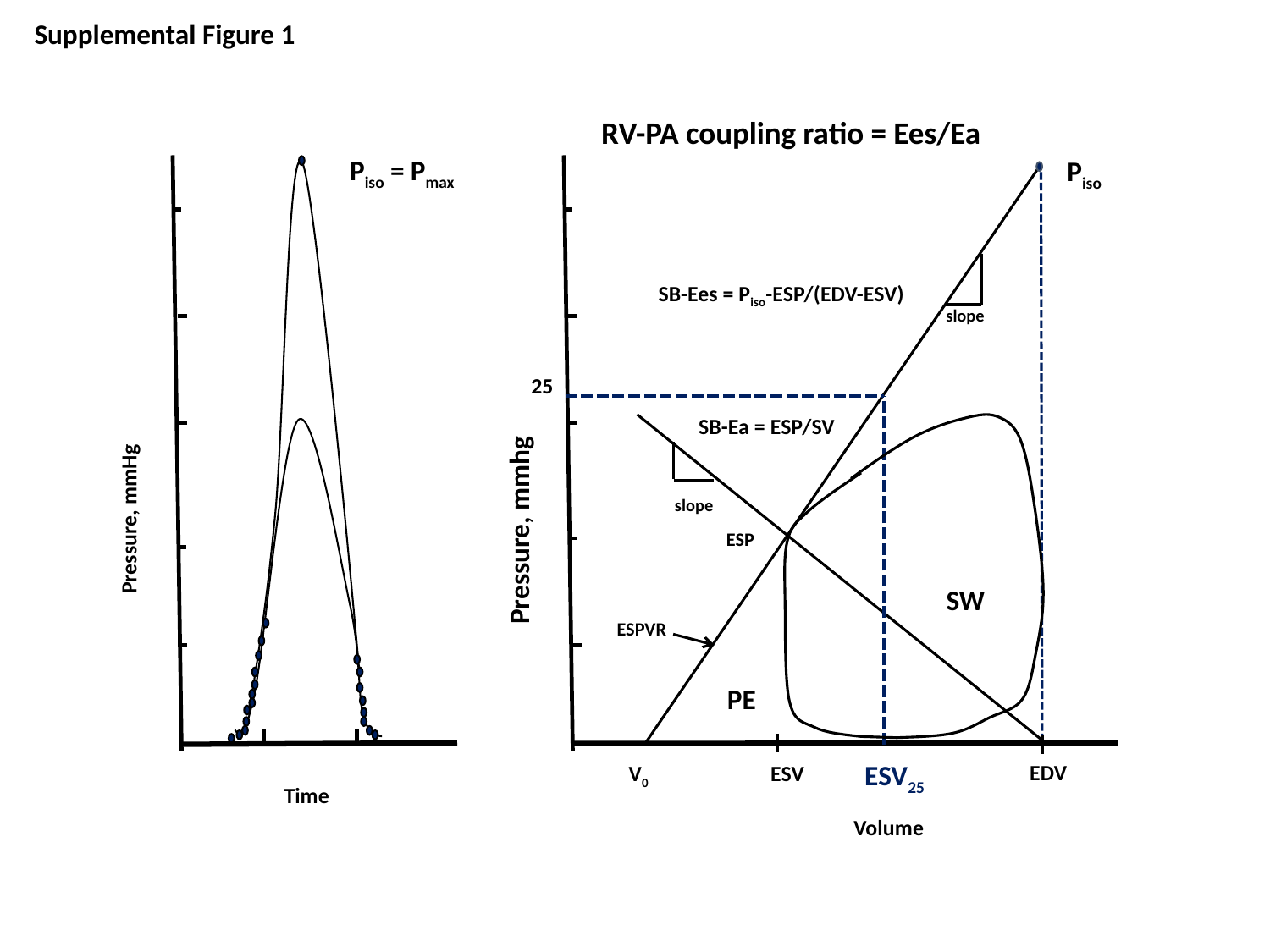

Supplemental Figure 1
RV-PA coupling ratio = Ees/Ea
Piso = Pmax
Piso
SB-Ees = Piso-ESP/(EDV-ESV)
slope
25
SB-Ea = ESP/SV
slope
Pressure, mmHg
Pressure, mmhg
ESP
SW
ESPVR
PE
ESV25
EDV
ESV
V0
Time
Volume

## Slide 2
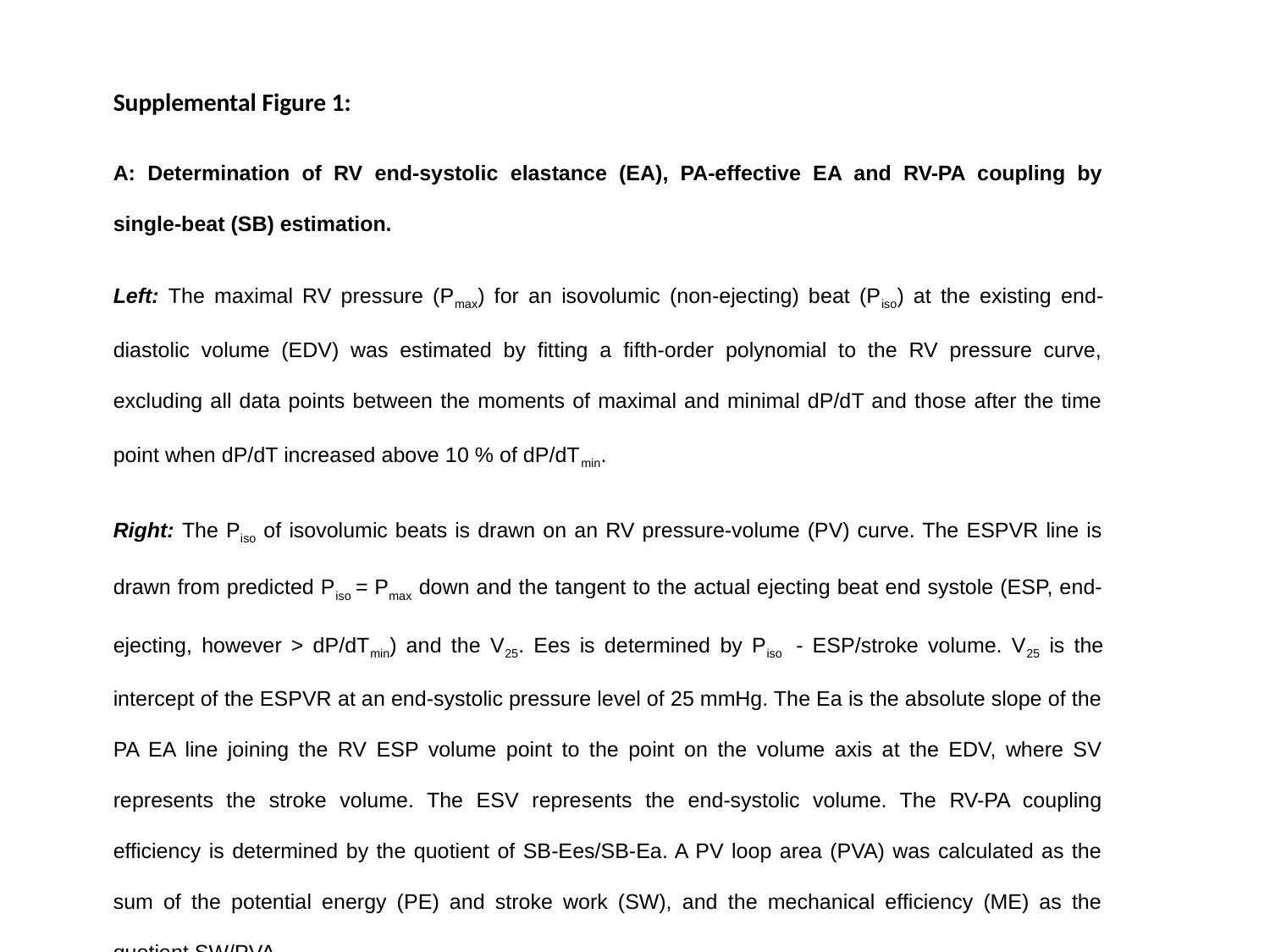

Supplemental Figure 1:
A: Determination of RV end-systolic elastance (EA), PA-effective EA and RV-PA coupling by single-beat (SB) estimation.
Left: The maximal RV pressure (Pmax) for an isovolumic (non-ejecting) beat (Piso) at the existing end-diastolic volume (EDV) was estimated by fitting a fifth-order polynomial to the RV pressure curve, excluding all data points between the moments of maximal and minimal dP/dT and those after the time point when dP/dT increased above 10 % of dP/dTmin.
Right: The Piso of isovolumic beats is drawn on an RV pressure-volume (PV) curve. The ESPVR line is drawn from predicted Piso = Pmax down and the tangent to the actual ejecting beat end systole (ESP, end-ejecting, however > dP/dTmin) and the V25. Ees is determined by Piso - ESP/stroke volume. V25 is the intercept of the ESPVR at an end-systolic pressure level of 25 mmHg. The Ea is the absolute slope of the PA EA line joining the RV ESP volume point to the point on the volume axis at the EDV, where SV represents the stroke volume. The ESV represents the end-systolic volume. The RV-PA coupling efficiency is determined by the quotient of SB-Ees/SB-Ea. A PV loop area (PVA) was calculated as the sum of the potential energy (PE) and stroke work (SW), and the mechanical efficiency (ME) as the quotient SW/PVA.

## Slide 3
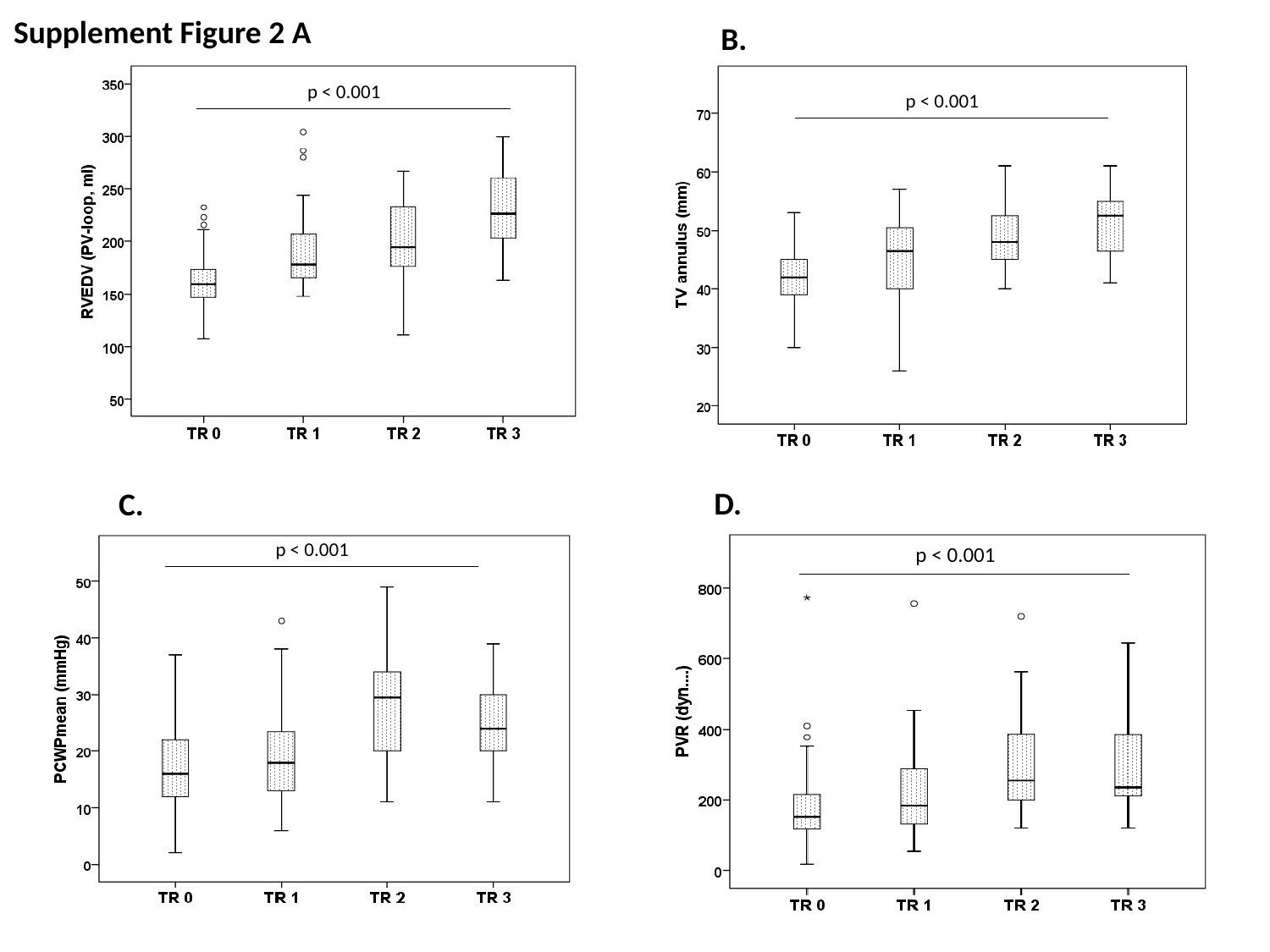

Supplement Figure 2 A
B.
p < 0.001
p < 0.001
D.
C.
p < 0.001
p < 0.001

## Slide 4
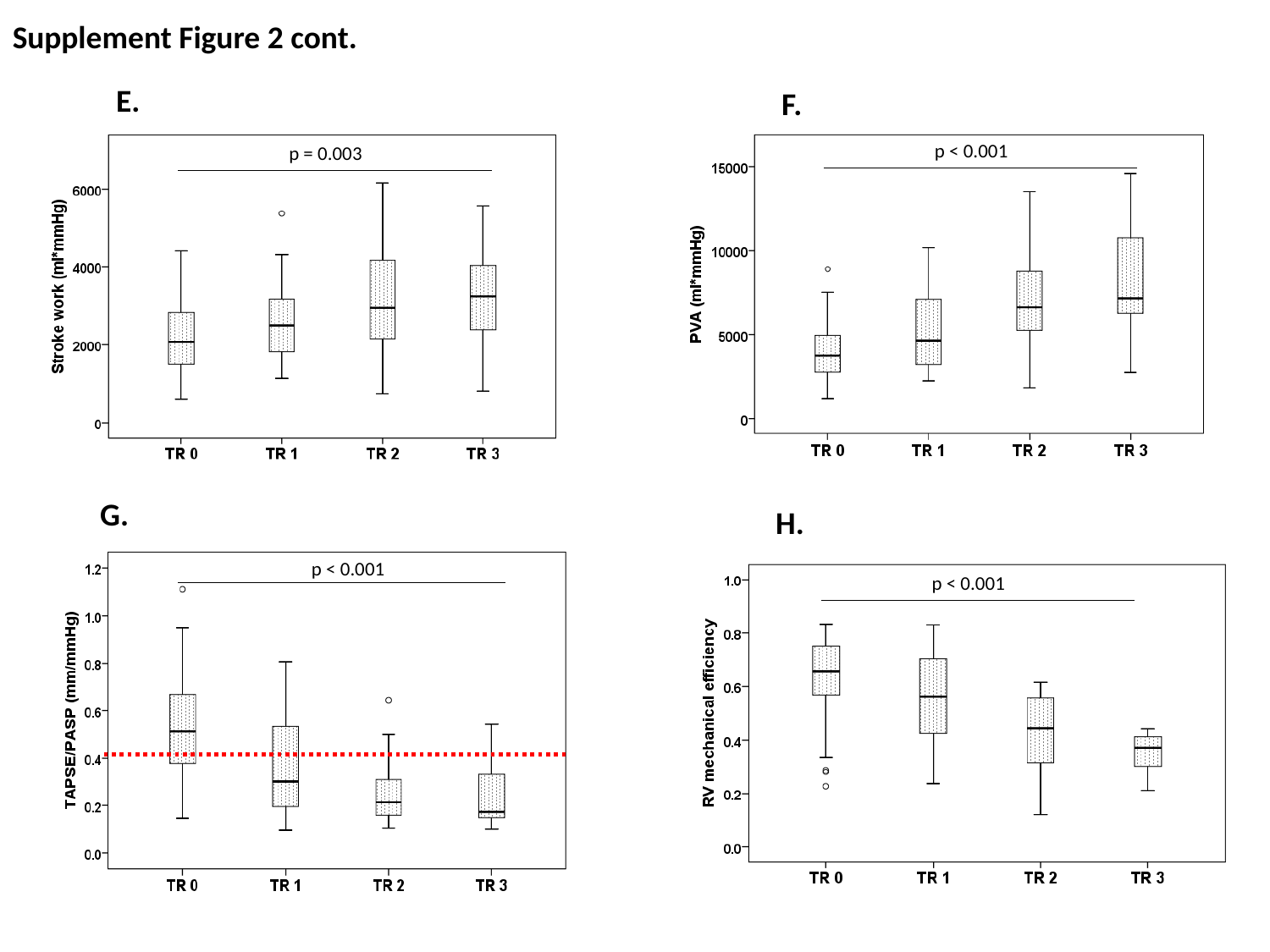

Supplement Figure 2 cont.
E.
F.
p = 0.003
p < 0.001
G.
H.
p < 0.001
p < 0.001

## Slide 5
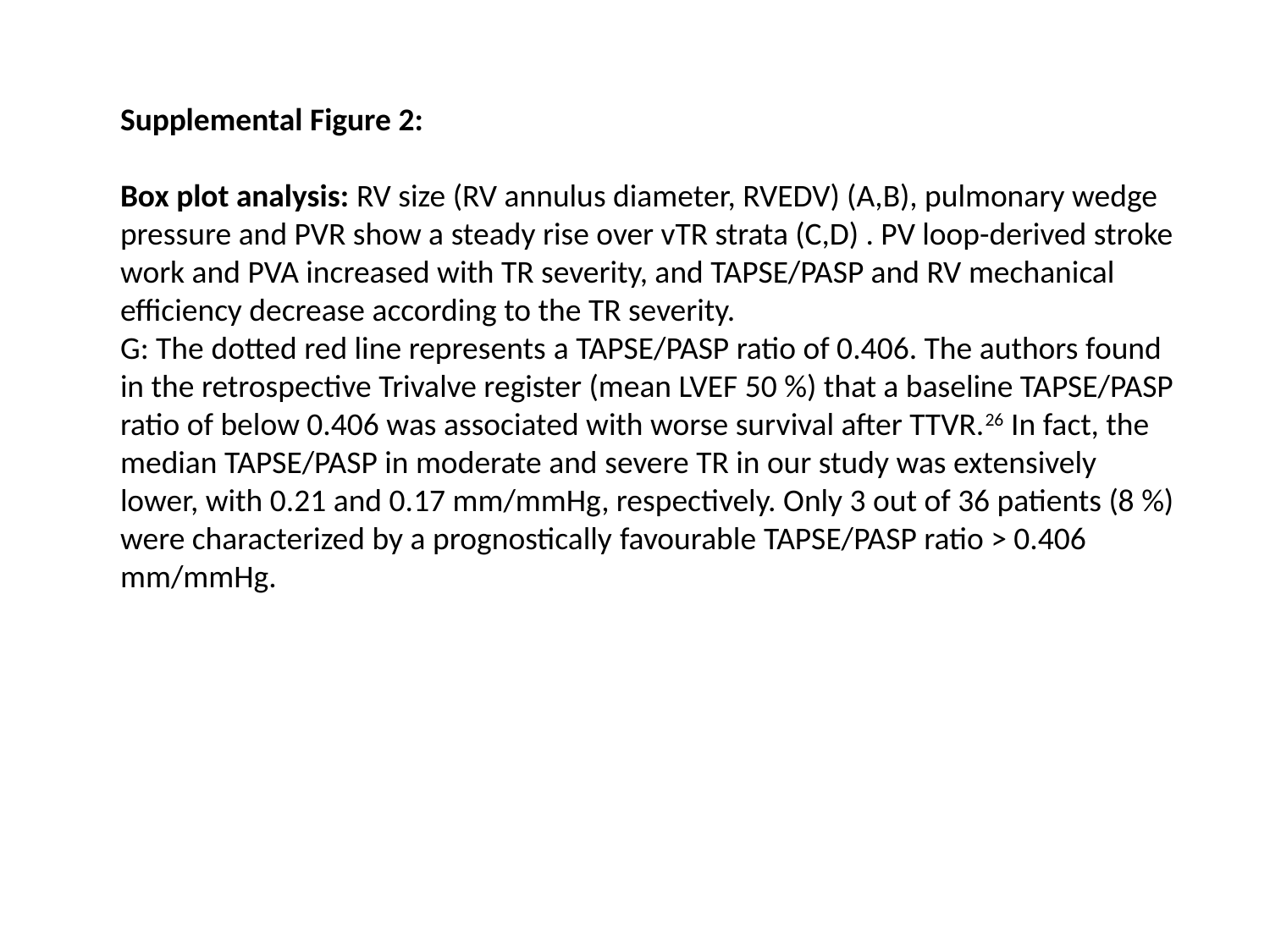

Supplemental Figure 2:
Box plot analysis: RV size (RV annulus diameter, RVEDV) (A,B), pulmonary wedge pressure and PVR show a steady rise over vTR strata (C,D) . PV loop-derived stroke work and PVA increased with TR severity, and TAPSE/PASP and RV mechanical efficiency decrease according to the TR severity.
G: The dotted red line represents a TAPSE/PASP ratio of 0.406. The authors found in the retrospective Trivalve register (mean LVEF 50 %) that a baseline TAPSE/PASP ratio of below 0.406 was associated with worse survival after TTVR.26 In fact, the median TAPSE/PASP in moderate and severe TR in our study was extensively lower, with 0.21 and 0.17 mm/mmHg, respectively. Only 3 out of 36 patients (8 %) were characterized by a prognostically favourable TAPSE/PASP ratio > 0.406 mm/mmHg.

## Slide 6
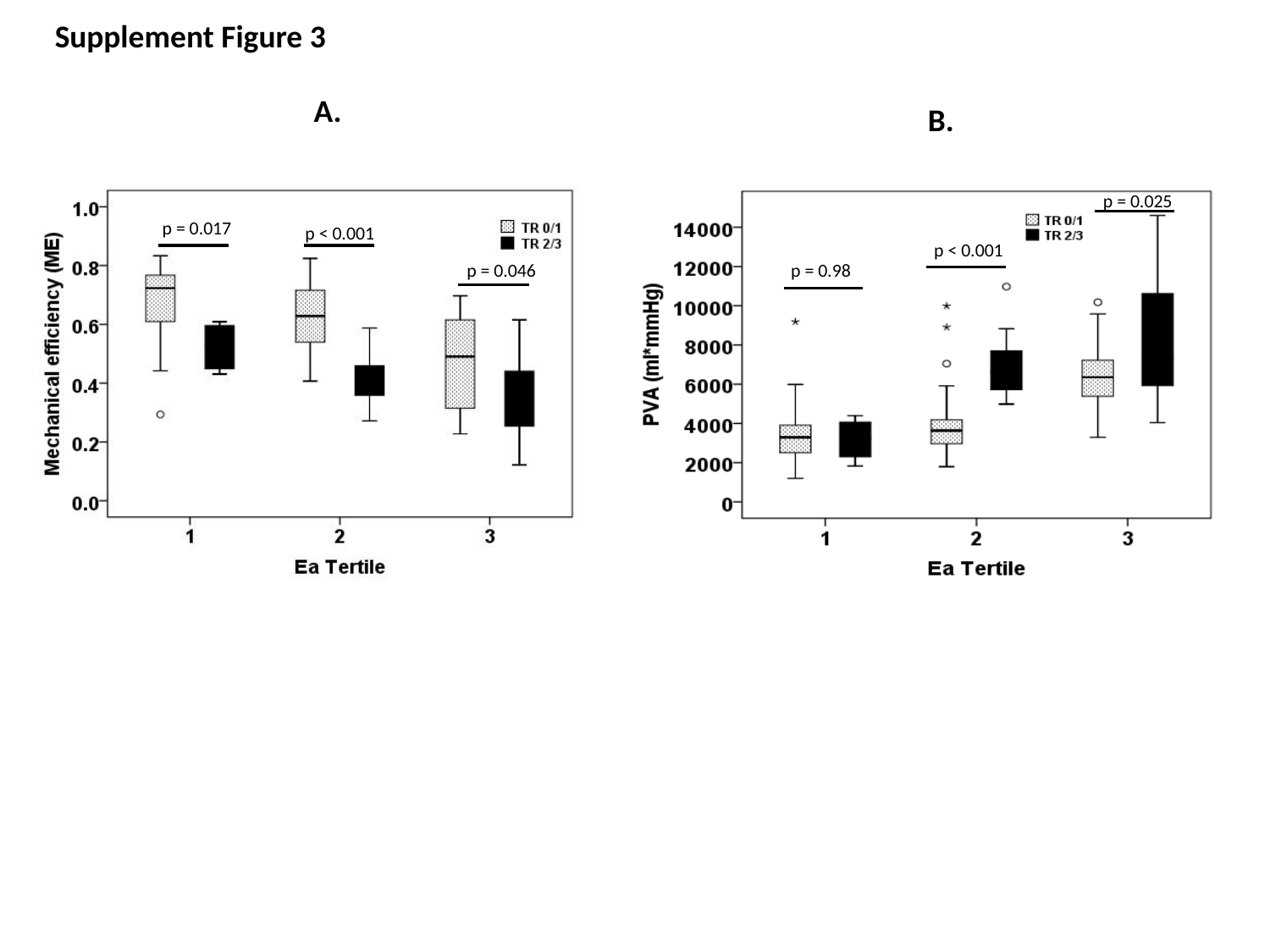

Supplement Figure 3
A.
B.
p = 0.025
p < 0.001
p = 0.98
p = 0.017
p < 0.001
p = 0.046

## Slide 7
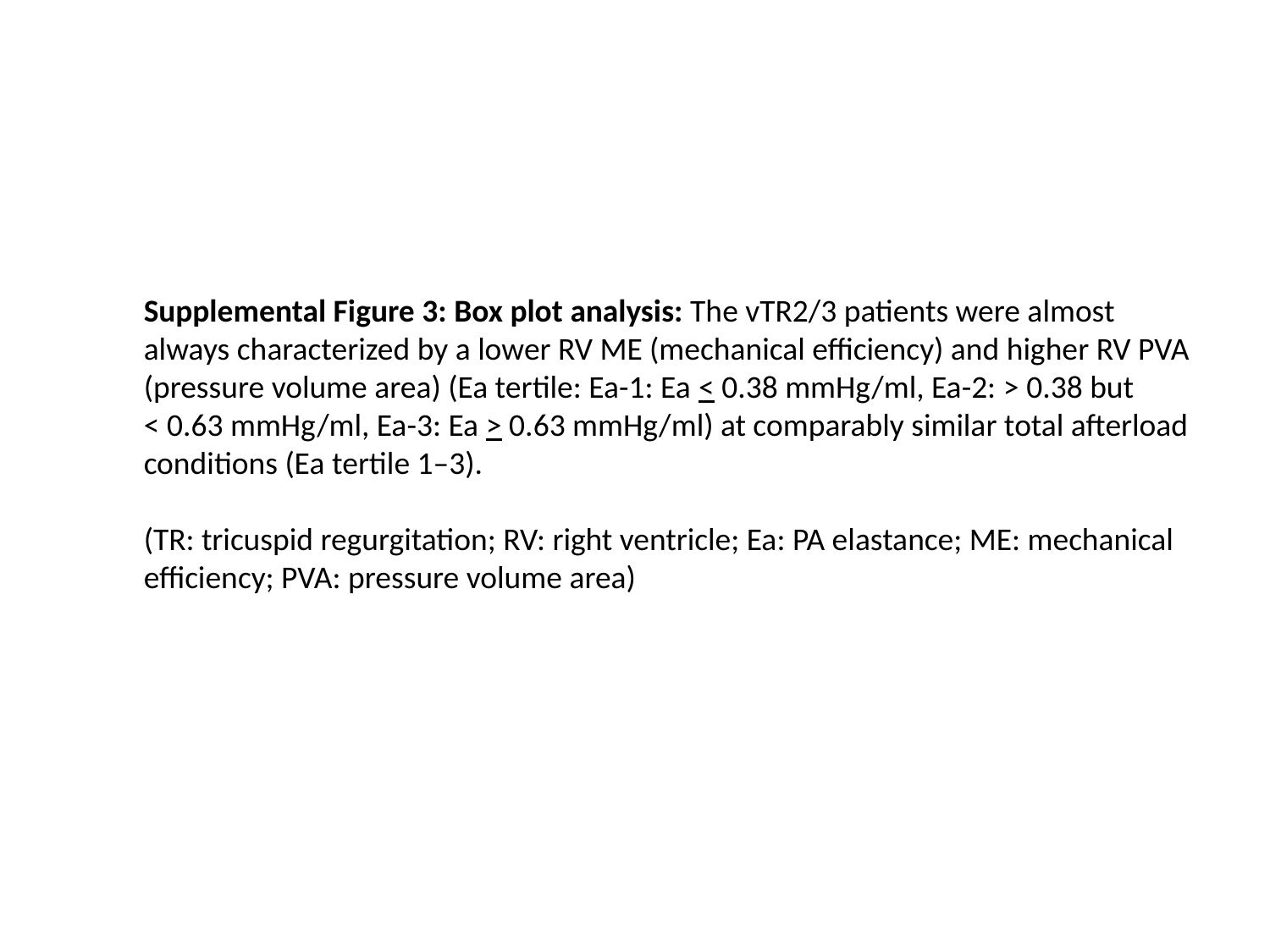

Supplemental Figure 3: Box plot analysis: The vTR2/3 patients were almost always characterized by a lower RV ME (mechanical efficiency) and higher RV PVA (pressure volume area) (Ea tertile: Ea-1: Ea < 0.38 mmHg/ml, Ea-2: > 0.38 but < 0.63 mmHg/ml, Ea-3: Ea > 0.63 mmHg/ml) at comparably similar total afterload conditions (Ea tertile 1–3).
(TR: tricuspid regurgitation; RV: right ventricle; Ea: PA elastance; ME: mechanical efficiency; PVA: pressure volume area)

## Slide 8
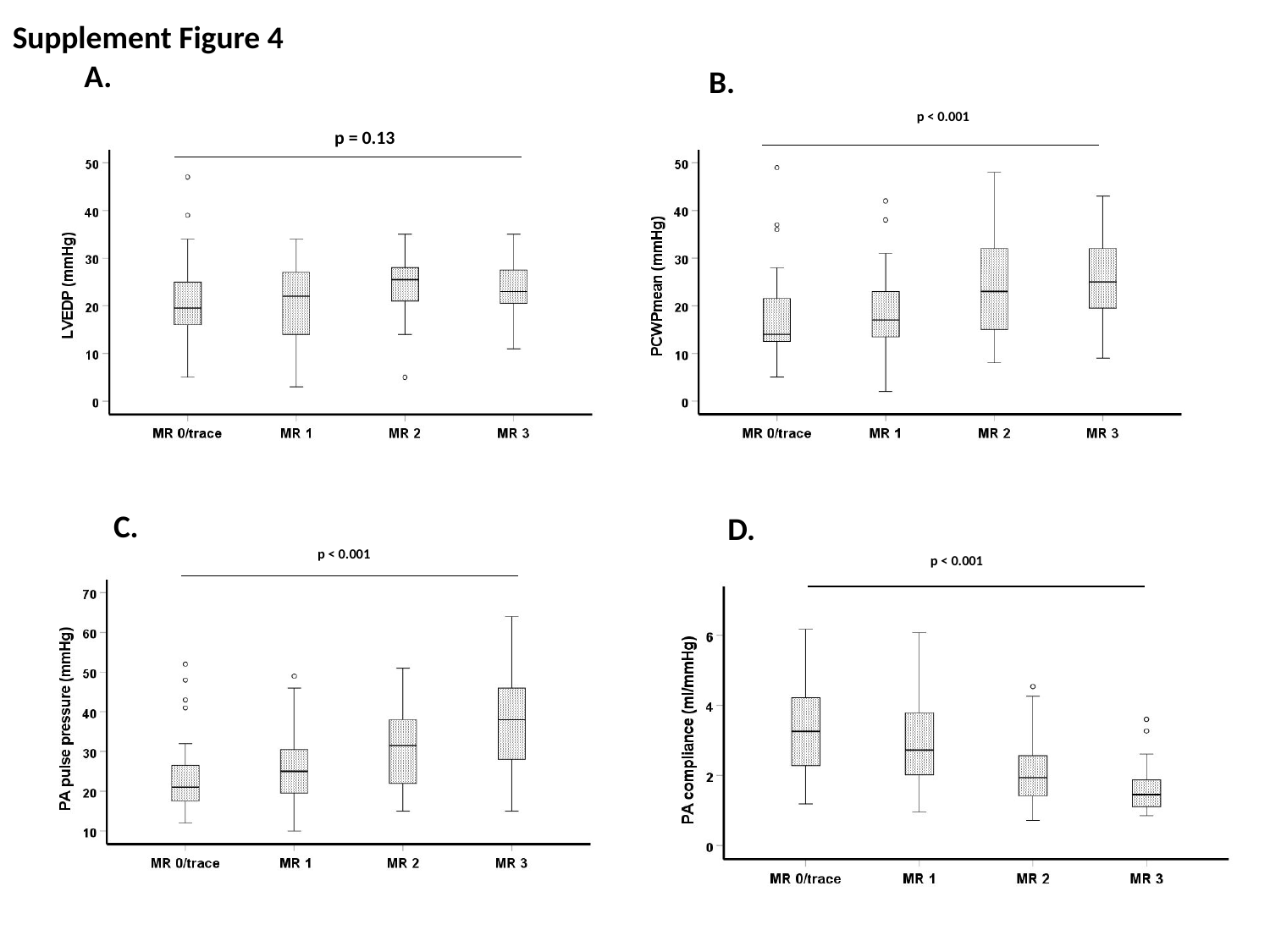

Supplement Figure 4
A.
B.
p < 0.001
p = 0.13
C.
p < 0.001
D.
p < 0.001

## Slide 9
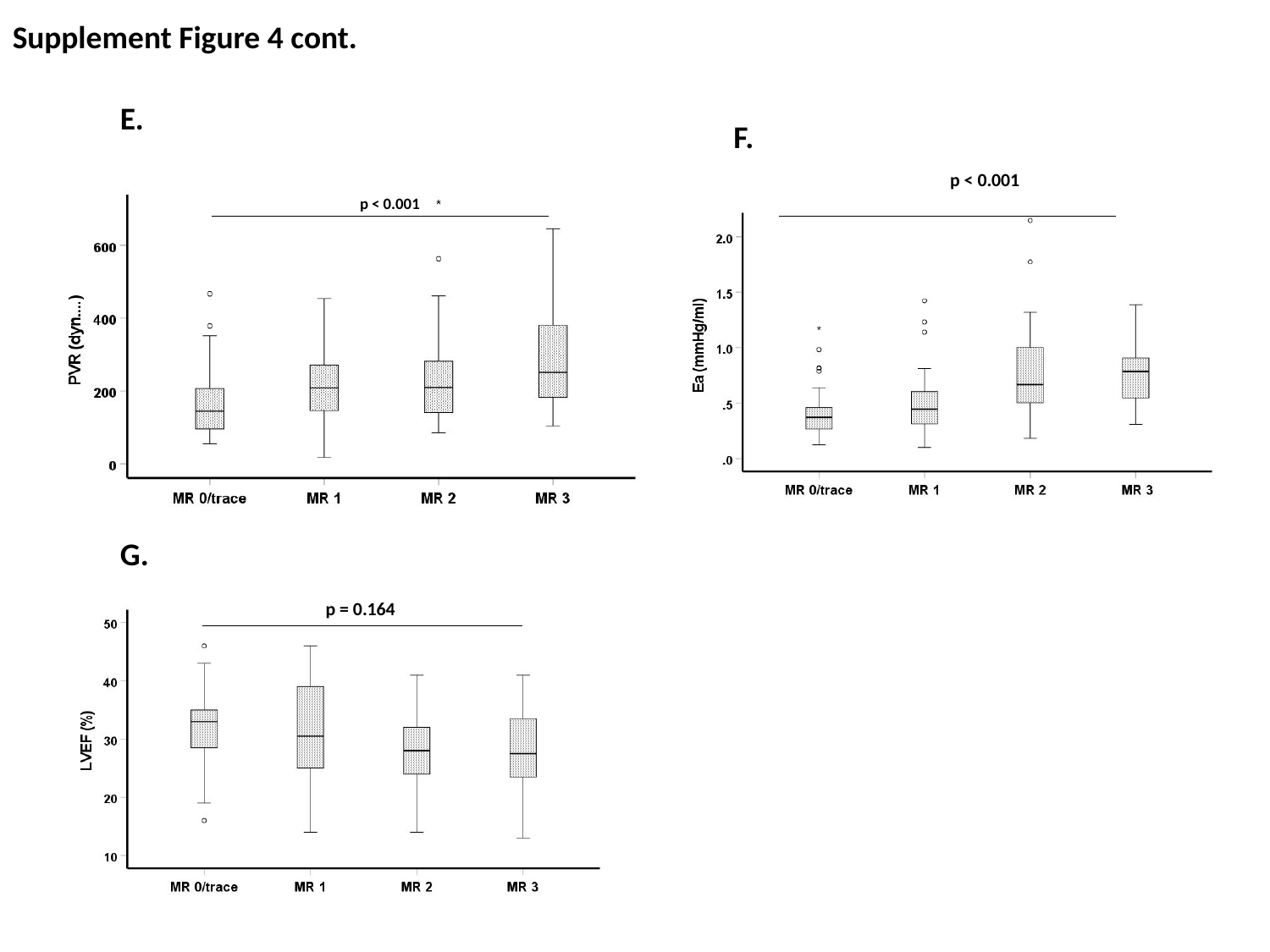

Supplement Figure 4 cont.
E.
F.
p < 0.001
p < 0.001
G.
p = 0.164

## Slide 10
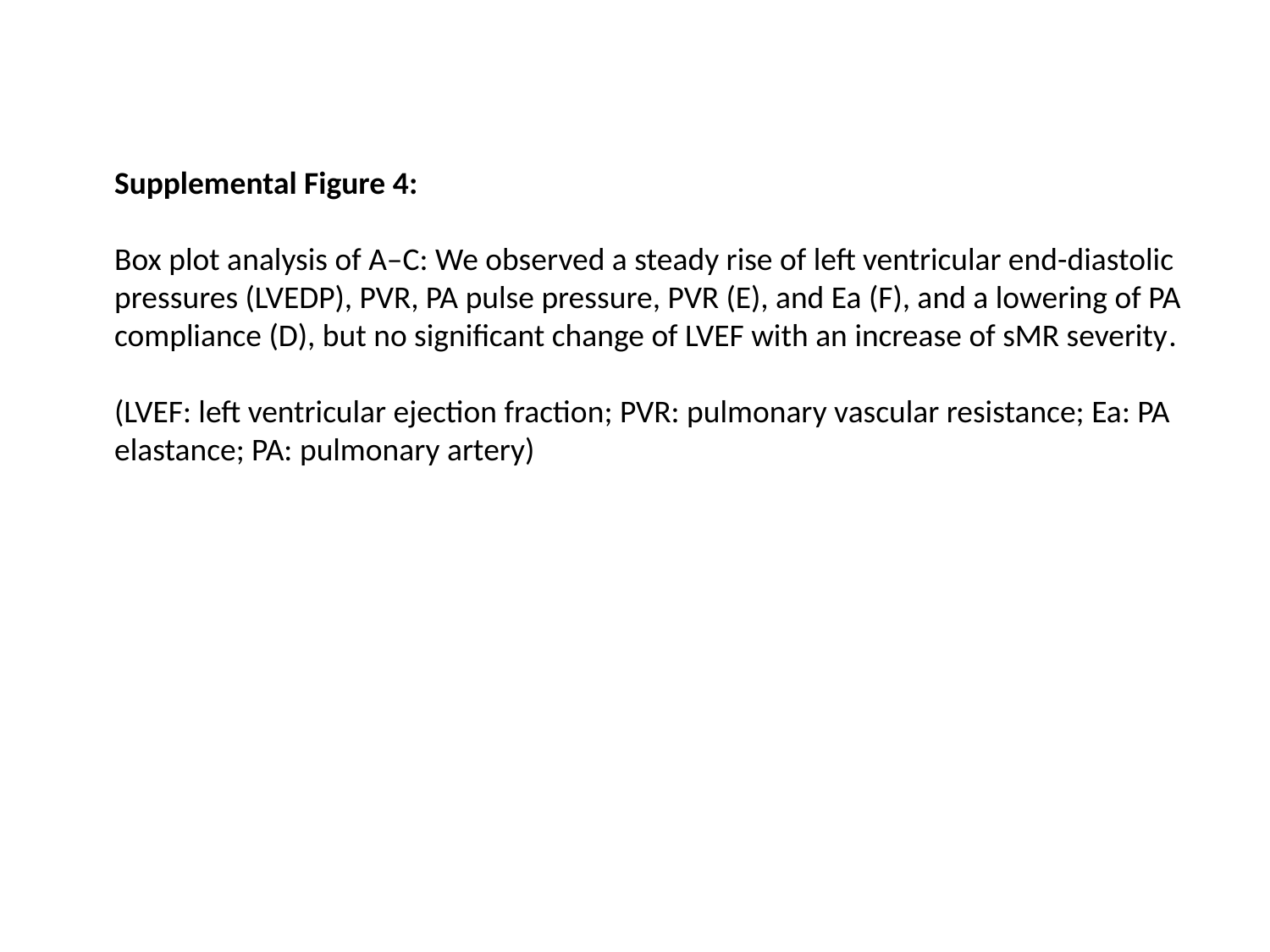

Supplemental Figure 4:
Box plot analysis of A–C: We observed a steady rise of left ventricular end-diastolic pressures (LVEDP), PVR, PA pulse pressure, PVR (E), and Ea (F), and a lowering of PA compliance (D), but no significant change of LVEF with an increase of sMR severity.
(LVEF: left ventricular ejection fraction; PVR: pulmonary vascular resistance; Ea: PA elastance; PA: pulmonary artery)

## Slide 11
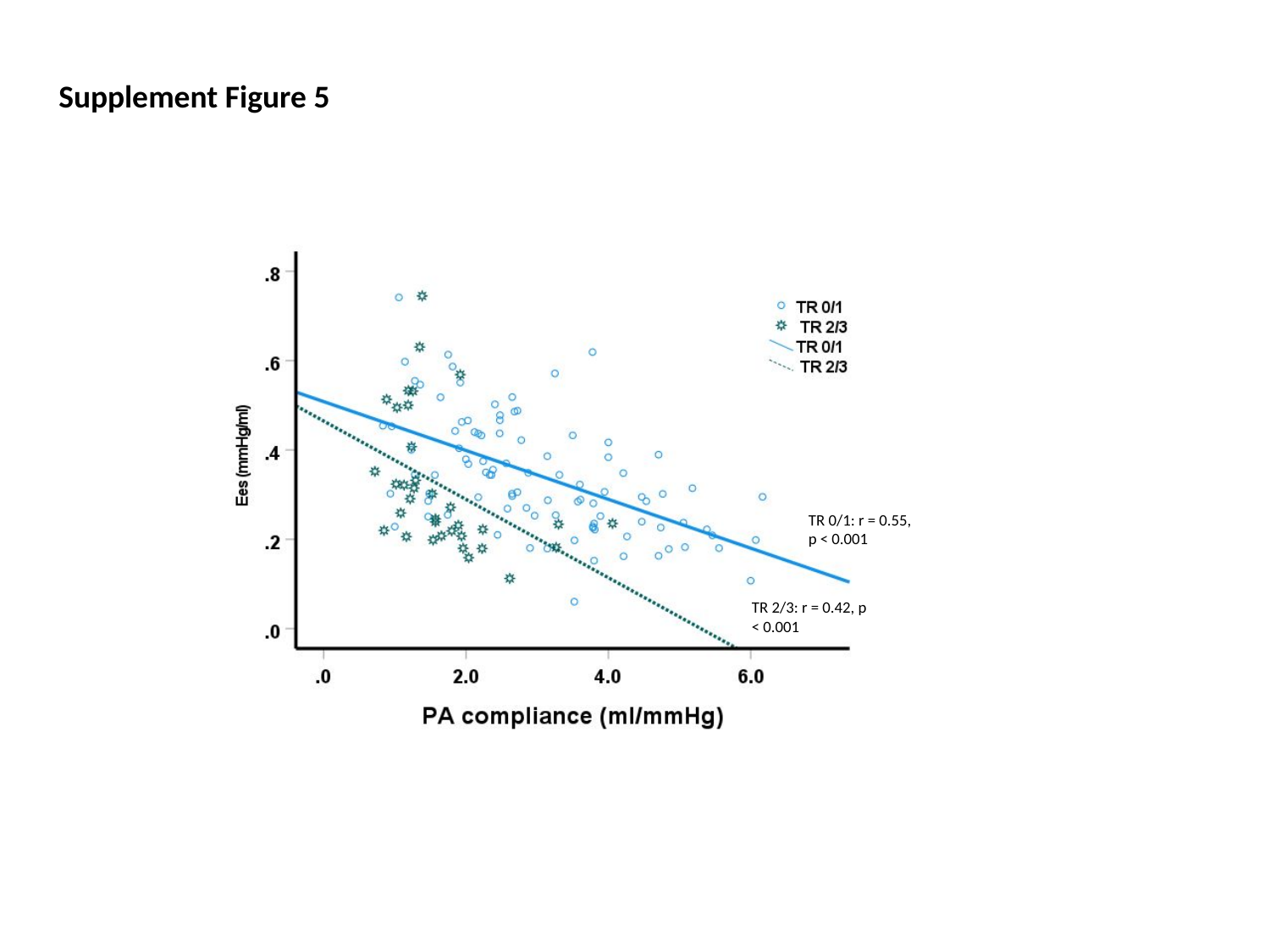

Supplement Figure 5
TR 0/1: r = 0.55, p < 0.001
TR 2/3: r = 0.42, p < 0.001

## Slide 12
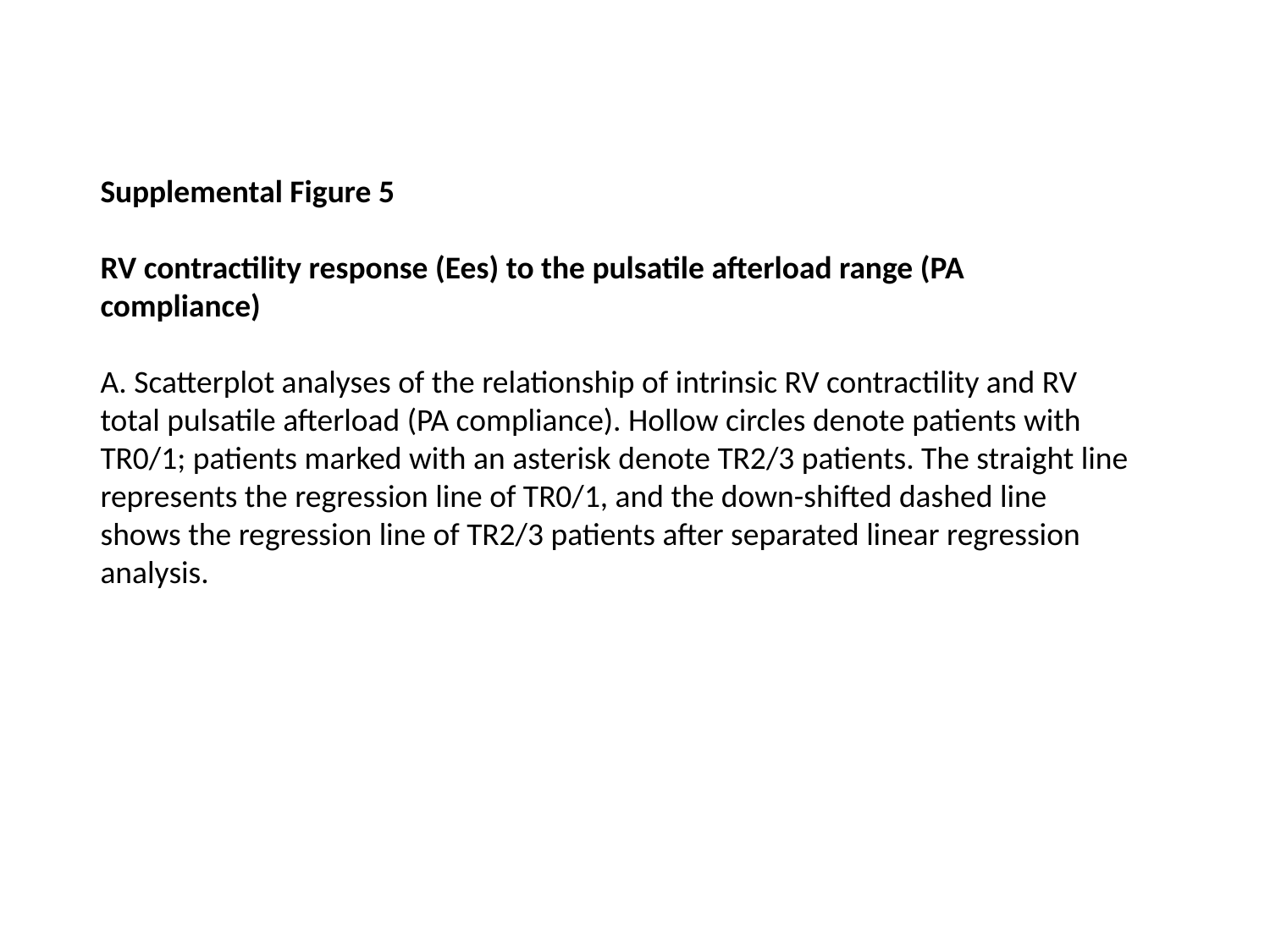

Supplemental Figure 5
RV contractility response (Ees) to the pulsatile afterload range (PA compliance)
A. Scatterplot analyses of the relationship of intrinsic RV contractility and RV total pulsatile afterload (PA compliance). Hollow circles denote patients with TR0/1; patients marked with an asterisk denote TR2/3 patients. The straight line represents the regression line of TR0/1, and the down-shifted dashed line shows the regression line of TR2/3 patients after separated linear regression analysis.
