# Supplementary material for: Interaction of secondary ventricular tricuspid regurgitation with RV in HFREF: an invasive pressure-volume loop study
Source: ESC Heart Fail. 2026 May 11;13(3):xvag134. doi: 10.1093/eschf/xvag134 (PMC13220961; doi:10.1093/eschf/xvag134)
Supplement: xvag134_Supplementary_Data [file xvag134_supplementary_data.zip › 25_supplemental table 1.docx]

**Supplemental Table 1: Baseline characteristics, LV function, RV afterload parameter according to the vTR severity**

|  | vTR-0  none/trace  (N = 66) | vTR-1  mild  (N = 32) | vTR-2  moderate  (N = 23) | vTR-3  Severe/  massive  (N = 13) | p for trend  TR 0–3 |
| --- | --- | --- | --- | --- | --- |
| Clinical characteristics | | | | | |
| Age (years) | 64 (57–73) | 71 (63–75) | 70 (63–75) | 70 (63–74) | 0.092 |
| Men (%) | 83 | 84.4 | 96 | 84.6 | 0.53 |
| ICM (%) | 44 | 56 | 61 | 61 | 0.31 |
| Creatinin (umol/l) | 77 (92–112) | 113 (87–136) | 134 (102–172) | 102 (92–196) | < 0.001 |
| NT-pro-BNP  (pg/ml) | 766 (429–1942) | 2286 (752–5962) | 3989 (1451–6275) | 6277 (3061–16828) | < 0.001 |
| PM/AICD/CRT (%) | 45.5 | 62.5 | 78 | 84.6 | 0.075 |
| Left ventricular morphology and function | | | | | |
| LVEDV (ml) | 212 (180–270) | 215 (171–286) | 202 (195–284) | 217 (194–278) | 0.89 |
| LV-EF (%) | 34 (26–36) | 29 (26–36) | 27 (24–33) | 23 (22–30) | 0.002 |
| LVEDP (mmHg) | 20 (14–27) | 22 (15–27) | 26 (22–29) | 23 (21–26) | 0.028 |
| MR 2/3 (%) | 18.2 | 50 | 69.6 | 77 | < 0.001 |
| LA volume (ml) | 74 (58–96) | 102 (80–128) | 103 (88–142) | 128 (99–142) | < 0.001 |
| RV afterload parameter (Swan-Ganz and PV loop catheter) | | | | | |
| PAmean  (mmHg) | 25 (20–31) | 29 (23–40) | 42 (33–50) | 37 (29–42) | < 0.001 |
| PAmean > 20 mmHg (%) | 73 | 84 | 95.5 | 100 | 0.025 |
| CpcPH (%) | 41 | 56 | 87 | 92 | < 0.001 |
| PA syst. (mmHg) | 37 (31–46) | 42 (35–58) | 66.5 (47–76) | 56 (48–68) | < 0.001 |
| PCWPmean  (mmHg) | 16 (12–22) | 18 (13–24) | 29.5 (20–34.5) | 24 (19–31) | < 0.001 |
| TPG  (mmHg) | 10 (7–12) | 12 (8–14) | 11.5 (10–16) | 14 (8–20) | 0.017 |
| PVR (dyn.) | 148 (117–214) | 184 (125–298) | 254 (197–404) | 235 (210–426) | < 0.001 |
| PA pulse pressure (mmHg) | 21 (18–27) | 27 (20–32) | 38 (27–47) | 33 (26–39) | < 0.001 |
| FW-SV (ml) | 66 (54–76) | 58 (50–66) | 52 (47–60) | 51 (45–61) | < 0.001 |
| PA compliance  (ml/mmHg) | 3.3 (2.2–4.3) | 2.4 (1.4–3.1) | 1.37 (1.2–1.8) | 1.57 (1.2–2.1) | < 0.001 |
| ESP  (mmHg) | 27 (21–39) | 36 (28–48) | 49 (38–62) | 50 (43–56) | < 0.001 |
| Ea  (mmHg/ml) | 0.39 (0.29–0.53) | 0.56 (0.38–0.79) | 0.81 (0.5–1.1) | 0.63 (0.55–0.9) | < 0.001 |

Values are median (25/75th percentiles)

ICM: ischemic cardiomyopathy; PM: pacemaker; AICD: automatic implantable cardioverter defibrillator; CRT: cardiac resynchronization therapy; LVEDV: left ventricular end-diastolic volume; LVEF: left ventricular ejection fraction; LVEDP: left ventricular end-diastolic pressure; MR: mitral regurgitation; TR: tricuspid regurgitation; LA: left atrial; PA: pulmonary artery pressure; CPcPH: combined post- and precapillary pulmonary hypertension; PH: pulmonary hypertension; PCWP: pulmonary capillary wedge pressure; TPG: trans-pulmonary gradient; PVR: pulmonary vascular resistance; FW-SV: forward stroke volume; ESP: end-systolic pressure; Ea: pulmonary arterial elastance
