# Supplementary material for: Interaction of secondary ventricular tricuspid regurgitation with RV in HFREF: an invasive pressure-volume loop study
Source: ESC Heart Fail. 2026 May 11;13(3):xvag134. doi: 10.1093/eschf/xvag134 (PMC13220961; doi:10.1093/eschf/xvag134)
Supplement: xvag134_Supplementary_Data [file xvag134_supplementary_data.zip › 26_supplemental table 2.docx]

| **Supplemental Table 2: PV loop-derived, RV echocardiographic, and MR data according to vTR severity** | vTR-0  none/trace  (N = 66) | vTR-1  mild  (N = 32) | vTR-2  moderate  (N = 23) | vTR-3  Severe/  massive  (N = 13) | p for trend  TR 0–3 |
| --- | --- | --- | --- | --- | --- |
| RV morphology and function (PV Loop Analysis, RV Echo, RV MRI) | | | | | |
| Ees  (mmHg/ml) | 0.31 (0.25–0.44) | 0.34 (0.23–0.44) | 0.27 (0.22–0.51) | 0.22 (0.18–0.33) | 0.161 |
| Ees/Ea | 0.84 (0.67–1.1) | 0.58 (0.41–1.0) | 0.48 (0.29–0.58) | 0.38 (0.28–0.45) | < 0.001 |
| RV uncoupled (Ees/Ea < 0.68) (%) | 26 | 60 | 82 | 100 | < 0.001 |
| Stroke work  (mmHg*ml) | 2085 (1497–2827) | 2506 (1817–3173) | 2958 (2136–4192) | 3239 (2233–4050) | 0.003 |
| PVA  (ml*mmHg) | 3737 (2779–4973) | 4646 (3191–7106) | 6629 (4987–8824) | 7145 (6105–10882) | < 0.001 |
| ME | 0.66 (0.57–0.75) | 0.56 (0.4–0.71) | 0.44 (0.28–0.56) | 0.37 (0.3–0.41) | < 0.001 |
| Eed (mmHg/ml) | 0.06 (0.03–0.07) | 0.05 (0.04–0.096) | 0.09 (0.06–0.14 | 0.14 (0.05–0.22) | 0.002 |
| Tau  (ms) | 65 (56–77) | 65 (57–72) | 71.5 (64–84) | 64 (58–81) | 0.14 |
| dp/dtmin  (mmHg/s) | -266(-348– -214) | -315 (-390– -221) | -373 (-459– -264) | -389 (-437– -326) | 0.002 |
| RA mean (mmHg) | 8 (6–11) | 8 (6–12) | 13 (11–16) | 16 (12–19) | < 0.001 |
| RVEDP  (mmHg) | 8 (6.75–11.8) | 8.8 (7–12.5) | 11.5 (9.8–14.6 | 14 (12–22) | 0.001 |
| RVEDV (PV loop, ml) | 159 (146–173) | 178 (165–208) | 194 (177–239) | 226 (202–260) | < 0.001 |
| TV annulus (mm) | 42 (39–45) | 46 (39–51) | 48 (45–53) | 52.5 (46–56) | < 0.001 |
| RVOT ED prox (mm) | 30 (27–34) | 33 (30–38) | 33.5 (30–45.5) | 42 (38–47.5) | < 0.001 |
| End-diastolic 4-chv RV area (cm^2^) | 21 (18–26) | 26 (20–31) | 32 (22–39) | 33.5 (31–41) | < 0.001 |
| TAPSE (mm) | 20 (16–22) | 14 (10–19) | 14 (12–16) | 11 (9–16) | < 0.001 |
| TAPSE/PASP  (mm/mmHg) | 0.51 (0.37–0.67) | 0.30 (0.19–0.53) | 0.21 (0.16–0.31) | 0.17 (0.14–0.34) | < 0.001 |
| TAPSE/PASP >0.406 mm/mmHg (N), % | (44) 67.7 | (12) 40 | (2) 9.1 | 1. 7.7 | < 0.001 |
| TAPSE/PASP  > 0.303  (N), % | (56) 84.8 | (15) 46.9 | (6) 26.1 | (4) 30.8 | < 0.001 |
| FAC (%) | 52 (42–58) | 40 (26–56) | 32 (26–41) | 31 (26–32) | < 0.001 |
| RV-EF (MRI, %) | 50 (43–53) | 41 (22–53) | 28 (21–30) | 22 (21–23) | 0.002 |
| RV mass (MRI, g) | 51  (46–58) | 54  (45–62) | 64  (61–72) | 71  (70–75) | 0.006 |
| RVmass/BSA  (MRI, g/m^2^) | 26  (24–28) | 26.6  (23–30) | 29  (26–31) | 38  (37–39) | 0.031 |
| RV M/V ratio  (MRI, g/ml) | 0.25  (0.27–37) | 0.28  (0.26–0.33) | 0.22  (0.21–0.24) | 0.19  (0.18–0.2) | 0.025 |

Values are median **(**25/75th percentiles**)**;

Ees: right ventricular end-systolic elastance; Ea: pulmonary arterial elastance; RV: right ventricular; PVA: pressure volume area; ME: mechanical efficiency; Eed: end-diastolic elastance RV; RA: right atrial; RVEDP: right ventricular end-diastolic pressure; RVEDV: right ventricular end-diastolic volume; TV: tricuspid valve; RVOT-ED: right ventricular outflow tract end-diastolic; 4-chv: 4 chamber view; RV-EF: right ventricular ejection fraction; FAC: RV fractional area change; TAPSE: tricuspid annular plane systolic excursion; PASP: systolic pulmonary arterial pressure; Tau: time constant for isovolumic relaxation; M: mass; V: volume.
