# Supplementary material for: Interaction of secondary ventricular tricuspid regurgitation with RV in HFREF: an invasive pressure-volume loop study
Source: ESC Heart Fail. 2026 May 11;13(3):xvag134. doi: 10.1093/eschf/xvag134 (PMC13220961; doi:10.1093/eschf/xvag134)
Supplement: xvag134_Supplementary_Data [file xvag134_supplementary_data.zip › 27_supplemental table 3.docx]

**Supplemental Table 3: The haemodynamic parameter PA compliance and Ees/Ea ratio, but not vTR2/3 per se, are independent predictors of all-cause mortality (haemodynamic model 1) in the Cox regression analysis.**

|  | **Univariate** | | **Multivariate** | |
| --- | --- | --- | --- | --- |
|  | **Exp (B) (95 % CI)** | **p** | **Exp (B) (95 % CI)** | **p** |
| **Age** | 1.07 (1.035–1.1) | < 0.001 | 1.035 (1.01–1.07) | 0.042 |
| **vTR (2–3)** | 1.83 (1.5–2.3) | < 0.001 |  |  |
| **vTR 0/1 vs. 2/3** | 3.8 (2.3–6.4) | < 0.001 |  |  |
| **PVR (dyn)** | 1.003 (1.002–1.005) | < 0.001 |  |  |
| **PA compliance (ml/mmHg)** | 0.52 (0.4–0.67) | < 0.001 | 0.69 (0.5–0.94) | 0.021 |
| **TPG (mmHg)** | 1.07 (1.02–1.12) | 0.003 |  |  |
| **LV-EF (%)** | 0.93 (0.9–0.96) | < 0.001 |  |  |
| **Ea (mmHg/ml)** | 3.8 (2.2–6.5) | < 0.001 |  |  |
| **Ees/Ea** | 0.06 (0.022–0.17) | < 0.001 | 0.24 (0.075–0.766) | 0.016 |
| **RVEDV (PV loop, ml)** | 1.01 (1.005–1.02) | < 0.001 |  |  |
| **RV-PVA (ml*mmHg)** | 2.53 (1.5–4.2) | < 0.001 |  |  |
| **sMR (0–3)** | 1.75 (1.38–2.23) | < 0.001 |  |  |

TR: TR 0–3 and TR 0/1 vs. 2/3 were separate from each other and included within the multivariate model: there was no significant change of the multivariate relevant prognostic parameter

PVA was excluded from MA because of the high collinearity with Ees/Ea

Ea was excluded from MA because of the high collinearity with PA compliance

TR 3 included 11 patients with severe TR and 2 patients with massive TR.

TR: tricuspid regurgitation; PVR: pulmonary vascular resistance; PA: pulmonary arterial; TPG: transpulmonary gradient; LV-EF: left ventricular ejection fraction; Ea: PA elastance; Ees: end-systolic elastance right ventricular; RVEDV: right ventricular end-diastolic volume; PVA: pressure volume area; MR: mitral regurgitation; RVEDV: right ventricular end-diastolic volume; sMR: secondary mitral regurgitation
