# Supplementary material for: Interaction of secondary ventricular tricuspid regurgitation with RV in HFREF: an invasive pressure-volume loop study
Source: ESC Heart Fail. 2026 May 11;13(3):xvag134. doi: 10.1093/eschf/xvag134 (PMC13220961; doi:10.1093/eschf/xvag134)
Supplement: xvag134_Supplementary_Data [file xvag134_supplementary_data.zip › 28_supplemental table 4.docx]

**Supplement Table 4: Using only the non-invasive parameter in the Cox regression analysis, vTR2/3 remains an independent predictor of all-cause mortality (non-invasive model 2).**

|  | **Univariate** | | **Multivariate** | |
| --- | --- | --- | --- | --- |
|  | **Exp (B) (95 % CI)** | **p** | **Exp (B) (95 % CI)** | **p** |
| **Age** | 1.07 (1.035–1.1) | < 0.001 | 1.05 (1.006–1.087) | 0.024 |
| **vTR 2/3** | 3.8 (2.3–6.4) | < 0.001 | 2.33 (1.1–4.7) | 0.019 |
| **Creatinin** | 1.01 (1.007–1014) | < 0.001 |  |  |
| **LA volume** | 1.015 (1.009–1.020) | < 0.001 |  |  |
| **NYHA** | 1.95 (1.28–2.98) | 0.002 | 6.1 (1.3–27.9) |  |
| **PA systolic (echo)** | 1.027 (1.013–1.041) | < 0.001 |  |  |
| **LVEF** | 0.93 (0.9–0.96) | < 0.001 |  |  |
| **TAPSE** | 0.87 (0.82–0.93 | < 0.001 | 0.9 (0.84–0.98) | 0.021 |
| **FAC** | 0.95 (0.93–0.98) | < 0.001 |  |  |
| **sMR (0–3)** | 1.75 (1.4–2.2) | < 0.001 |  |  |

TR: tricuspid regurgitation; LA: left atrial; NYHA: New York Heart Association; PA: pulmonary arterial; LVEF: left ventricular ejection fraction; sMR: secondary mitral regurgitation; FAC: fractional area change; TAPSE: tricuspid annular plane systolic excursion
