# Supplementary material for: Interaction of secondary ventricular tricuspid regurgitation with RV in HFREF: an invasive pressure-volume loop study
Source: ESC Heart Fail. 2026 May 11;13(3):xvag134. doi: 10.1093/eschf/xvag134 (PMC13220961; doi:10.1093/eschf/xvag134)
Supplement: xvag134_Supplementary_Data [file xvag134_supplementary_data.zip › 40_Sensitivity Table S1 Group 1 baseline characteristics.docx]

| Baseline Characteristics | Measure (N = 111) |
| --- | --- |
| Age (years) | **66.5 (59-73)** |
| Men (%) | **85** |
| NYHA III (%) | **86.4 (77)** |
| ICM (%) | **56** |
| Permanent/persistent atrial fibrillation  (%) | **5** |
| Diuretics (%) | **86** |
| ACE Inhibitors/ARBs/ ARNI (%) | **95** |
| B-Blockers (%) | **97** |
| Aldosteron-I. (%) | **52** |
| *LV Parameter* |  |
| LV-EF (ml) | **32 (26–32)** |
| LVEDV (ml) | **211 (180–261)** |
| LA volume (ml) | **88 (65–110)** |
| MR 2/3, N (%) | **39 (35.5)** |
| *Swan-Ganz catheter* |  |
| PA mean (mmHg) | **28 (22–40)** |
| PCWP mean (mmHg | **17 (13-27)** |
| PVR (dyn.) | **191 (122–258)** |
| PA compliance (ml/mmHg) | **2.56 (1.57-3.8)** |
| *TR (echo)* |  |
| TR 0/trace N (%) | **62 (55.9)** |
| TR mild (I) N (%) | **26 (23.4)** |
| TR moderate (II) N (%) | **18 (16.2)** |
| TR severe/massive (III) N (%) | **5 (4.5)** |

**M**edian (25th–75th percentile)

ACE-I: angiotensin converting enzyme inhibitor; AT: angiotension receptor; MRA: mineralocorticoid receptor antagonist; ICM: ischemic cardiomyopathy; PA_mean_: mean pulmonary arterial pressure; PH: pulmonary hypertension; LVEDV: left ventricular end-diastolic volume; LVEF: LV ejection fraction; LVEDP: **Left ventricular** end-diastolic pressure; MR: mitral regurgitation; LA: left atrium; PVR: pulmonary vascular resistance; PCWP: pulmonary capillary wedge pressure; TR: tricuspid regurgitation
