# Supplementary material for: Interaction of secondary ventricular tricuspid regurgitation with RV in HFREF: an invasive pressure-volume loop study
Source: ESC Heart Fail. 2026 May 11;13(3):xvag134. doi: 10.1093/eschf/xvag134 (PMC13220961; doi:10.1093/eschf/xvag134)
Supplement: xvag134_Supplementary_Data [file xvag134_supplementary_data.zip › 42_Sensitivity Table S1 Group 2 baseline characteristics.docx]

| Baseline Characteristics | Measure (N = 23) |
| --- | --- |
| Age (years) | **71 (64–75)** |
| Men (%) | **60** |
| NYHA III (%) | **65 (75)** |
| ICM (%) | **49** |
| Permanent/persistent atrial fibrillation  (%) | **3** |
| Diuretics (%) | **100** |
| ACE Inhibitors/ARBs/ ARNI (%) | **97** |
| B-Blockers (%) | **89** |
| Aldosteron-I. (%) | **48** |
| *LV Parameter* |  |
| LV-EF (ml) | **24 (21–28)** |
| LVEDV (ml) | **262 (1214–320)** |
| LA volume (ml) | **91 (67–120)** |
| MR 2/3, N (%) | **54 (41)** |
| *Swan-Ganz catheter* |  |
| PA mean (mmHg) | **32 (25–39)** |
| PCWP mean (mmHg | **22 (13–25)** |
| PVR (dyn.) | **214 (149–387)** |
| PA compliance (ml/mmHg) | **2.0 (1.2–2.8)** |
| *TR (echo)* |  |
| TR 0/trace N (%) | **4 (17)** |
| TR mild (I) N (%) | **6 (26)** |
| TR moderate (II) N (%) | **5 (22)** |
| TR severe/massive (III) N (%) | **6/2 (25/10)** |

**M**edian (25–75th percentile)

ACE-I: angiotensin converting enzyme inhibitor; AT: angiotension receptor; MRA: mineralocorticoid receptor antagonist; ICM: ischemic cardiomyopathy; PA_mean_: mean pulmonary arterial pressure; PH: pulmonary hypertension; LVEDV: left ventricular end-diastolic volume; LVEF: LV ejection fraction; LVEDP: left ventricular end-diastolic pressure; MR: mitral regurgitation; LA: left atrium; PVR: pulmonary vascular resistance; PCWP: pulmonary capillary wedge pressure; TR: tricuspid regurgitation
