# Supplementary material for: Interaction of secondary ventricular tricuspid regurgitation with RV in HFREF: an invasive pressure-volume loop study
Source: ESC Heart Fail. 2026 May 11;13(3):xvag134. doi: 10.1093/eschf/xvag134 (PMC13220961; doi:10.1093/eschf/xvag134)
Supplement: xvag134_Supplementary_Data [file xvag134_supplementary_data.zip › 44_Sensitivity Table S2 Group 1 binary logistic regression.docx]

**Sensitivity Analysis Group 1: Table S2: Lowered PA compliance and RV Ees/Ea ratios are independently associated with the occurrence of vTR2/3 in patients with HFREF in multivariate binary logistic regression analysis.**

|  | **Univariate** | | **Multivariate** | |
| --- | --- | --- | --- | --- |
|  | **Odds Ratio (95 % CI)** | **p** | **Odds Ratio (95 % CI)** | **p** |
| **PM/AICD/CRT (%)** | 2.09 (0.78–5.6) | 0.14 |  |  |
| **PCWP mean (mmhg)** | 1.12 (1.06–1.2) | < 0.001 |  |  |
| **PVR (dyn)** | 1.005 (1.002–1.009) | 0.005 |  |  |
| **PA compliance (ml/mmHg)** | 0.16 (0.065–0.4) | < 0.001 | 0.28 (0.11–0.74) | 0.01 |
| **LV-EF (%)** | 0.95 (0.89–1.02) | 0.15 |  |  |
| **LA volume diastolic (ml)** | 1.033 (1.015–1.031) | < 0.001 |  |  |
| **Ees/Ea** | 0.005 (0.001–0.064) | < 0-001 | 0.049 (0.002–1.014) | 0.053 |
| **sMR (0-3)** | 2.7 (1.6–4.5) | < 0.001 | 1.5 (0.8–2.8) | 1.6 |
| **RVEDV (ml, PV loop)** | 1.024 (1.01–1.04) | < 0.001 |  |  |

TR: tricuspid regurgitation; ICM: ischemic cardiomyopathy; PM: pacemaker; AICD: automatic implantable cardioverter defibrillator; CRT: cardiac resynchronization therapy; PA: pulmonary artery pressure; PCWP: pulmonary capillary wedge pressure; PVR: pulmonary vascular resistance; LV-EF: left ventricular ejection fraction; LVEDP: left ventricular end-diastolic pressure; LA: left atrial; Ea: PA elastance; Ees: end-systolic elastance of the right ventricle; FAC: right ventricular fractional area change; TAPSE: tricuspid annular plane systolic excursion; PASP: systolic pulmonary arterial pressure; sMR: secondary mitral regurgiatation; RVEDV: right ventricular end-diastolic volume; PV: pressure volume
