# Supplementary material for: Interaction of secondary ventricular tricuspid regurgitation with RV in HFREF: an invasive pressure-volume loop study
Source: ESC Heart Fail. 2026 May 11;13(3):xvag134. doi: 10.1093/eschf/xvag134 (PMC13220961; doi:10.1093/eschf/xvag134)
Supplement: xvag134_Supplementary_Data [file xvag134_supplementary_data.zip › 46_Sensitivity Table S2 Group 2 binary logistic regression.docx]

**Sensitivity Analysis Group 2 (n = 23) Table S2: Only an increased PCWP shows a strong trend for an association with an occurrence of vTR2/3 in multivariate binary logistic regression analysis.**

|  | **Univariate** | | **Multivariate** | |
| --- | --- | --- | --- | --- |
|  | **Odds Ratio (95 % CI)** | **p** | **Odds Ratio (95 % CI)** | **p** |
| **PCWP mean (mmhg)** | 1.15 (0.99–1.33) | 0.052 | 1.2 (0.99–1.4) | 0.051 |
| **PVR (dyn)** | 1.005 (0.99–1.01) | 0.124 |  |  |
| **PA compliance (ml/mmHg)** | 0.53 (0.2–1.4) | 0.19 |  |  |
| **LV-EF (%)** | 0.8 (0.66–1.01) | 0.07 |  |  |
| **LA volume diastolic (ml)** | 0.98 (0.96–1.008) | 0.18 |  |  |
| **RV Ees/Ea** | 0.01 (0.00–0.89) | 0.044 |  |  |
| **MR (0-3)** | 1.46 (0.69–3.1) | 0.3 |  |  |
| **RVEDV (ml, PV loop)** | 1.036 (1.002–1.07) | 0.036 |  |  |

TR: tricuspid regurgitation; ICM: ischemic cardiomyopathy; PM: pacemaker; AICD: automatic implantable cardioverter defibrillator; CRT: cardiac resynchronization therapy; PA: pulmonary artery pressure; PCWP: pulmonary capillary wedge pressure; PVR: pulmonary vascular resistance; LV-EF: left ventricular ejection fraction; LVEDP: left ventricular end-diastolic pressure; LA: left atrial; Ea: PA elastance; Ees: end-systolic elastance of the right ventricle; FAC: right ventricular fractional area change; TAPSE: tricuspid annular plane systolic excursion; PASP: systolic pulmonary arterial pressure; MR: mitral regurgiatation; RVEDV: right ventricular end-diastolic volume; PV: pressure volume
