# Supplementary material for: Interaction of secondary ventricular tricuspid regurgitation with RV in HFREF: an invasive pressure-volume loop study
Source: ESC Heart Fail. 2026 May 11;13(3):xvag134. doi: 10.1093/eschf/xvag134 (PMC13220961; doi:10.1093/eschf/xvag134)
Supplement: xvag134_Supplementary_Data [file xvag134_supplementary_data.zip › 48_Sensitivity Table S3 Group1.docx]

**Sensitivity analysis: Group 1 (n = 111) Table S3: A higher PCWP and the occurrence of an sMR2/3 are independently associated with a PA compliance < 0.6 mmHG/ml in a multivariate binary logistic regression analysis.**

|  | **Univariate** | | **Multivariate** | |
| --- | --- | --- | --- | --- |
|  | **Odds Ratio (95 % CI)** | **p** | **Odds Ratio (95 % CI)** | **p** |
| **LVEDP (mmHg)** | 1.1 (1.07–1.2) | < 0.001 |  |  |
| **PCWP (mmHg)** | 1.23 (1.14–1.3) | < 0.001 | 1.2 (1.1–1.3) |  |
| **LA size (ml)** | 1.022 (1.008–1.03) | 0.002 |  |  |
| **LVEF (%)** | 0.94 (0.89–0.99) | 0.033 |  |  |
| **Age (years** | 1.05 (1.008–1.1) | 0.02 |  |  |
| **sMR 2/3** | 7.38 (3–18) | < 0.001 | 5.9 (1.8–18.9) |  |

LVEDP: left ventricular end-diastolic pressure; PCWP: pulmonary capillary wedge pressure; LA: left atrial; LVEF: left ventricular ejection fraction; LVEDV: left ventricular end-diastolic pressure volume; sMR: secondary mitral regurgitation
