# Supplementary material for: Interaction of secondary ventricular tricuspid regurgitation with RV in HFREF: an invasive pressure-volume loop study
Source: ESC Heart Fail. 2026 May 11;13(3):xvag134. doi: 10.1093/eschf/xvag134 (PMC13220961; doi:10.1093/eschf/xvag134)
Supplement: xvag134_Supplementary_Data [file xvag134_supplementary_data.zip › 50_Sensitivity Table S3 Group2.docx]

**Sensitivity analysis: Group 2 (n = 23) Table S3: A higher age and the occurrence of an sMR are independently associated with a PA compliance < 0.6 ml/mmHg in a multivariate binary logistic regression analysis.**

|  | **Univariate** | | **Multivariate** | |
| --- | --- | --- | --- | --- |
|  | **Odds Ratio (95 % CI)** | **p** | **Odds Ratio (95 % CI)** | **p** |
| **LVEDP (mmHg)** | 1.18 (0.98-1–4) | 0.081 |  |  |
| **PCWP (mmHg)** | 1.26 (1.04–1.53) | 0.019 |  |  |
| **LA size (ml)** | 1.005 (0.98–1.02) | 0.7 |  |  |
| **LVEF (%)** | 0.9 (0.84–1.1) | 0.6 |  |  |
| **Age (years** | 1.13 (0.99–1.3) | 0.055 | 1.25 (1.01–1.5) | 0.039 |
| **sMR 1/2/3** | 8.4 (0.71–100) | 0.093 | 41 (1.1–1469) | 0.04 |

LVEDP: left ventricular end-diastolic pressure; PCWP: pulmonary capillary wedge pressure; LA: left atrial; LVEF: left ventricular ejection fraction; LVEDV: left ventricular end-diastolic pressure volume; sMR: secondary mitral regurgitation
