# Supplementary material for: Interaction of secondary ventricular tricuspid regurgitation with RV in HFREF: an invasive pressure-volume loop study
Source: ESC Heart Fail. 2026 May 11;13(3):xvag134. doi: 10.1093/eschf/xvag134 (PMC13220961; doi:10.1093/eschf/xvag134)
Supplement: xvag134_Supplementary_Data [file xvag134_supplementary_data.zip › 52_Sensitivity Table S4 Group1.docx]

**Sensitivity analysis Group 1 (n = 111): Table S4: A lower LVEF and a larger RV are independently associated with a pronounced RV-PA uncoupling (EesEa < 0.6) in multivariate binary logistic regression analysis.**

|  | **Univariate** | | **Multivariate** | |
| --- | --- | --- | --- | --- |
|  | **Odds Ratio (95 % CI)** | **p** | **Odds Ratio (95 % CI)** | **p** |
| **LVEDP (mmHg)** | 1.08 (1.02–1.1) | 0.006 |  |  |
| **PCWP (mmHg)** | 1.09 (1.04–1.1) | < 0.001 |  |  |
| **TPG (mmHg)** | 1.1 (1.03–1.2) | 0.01 |  |  |
| **LVEF (%)** | 0.9 (0.8–0.96) | 0.001 | 0.877 (0.79–0.97) | 0.007 |
| **RVEDV (ml)** | 1.04 (1.02–1.05) | < 0.001 | 1.044 (1.09–1.06) | <0.001 |
| **Age (years)** | 1.017 (0.97–1.06) | 0.4 |  |  |
| **PA compliance (ml/mmHg)** | 0.29 (0.17–0.48) | < 0.001 | 0.5 (0.29–1.05) | 0.06 |
| **PVR (dyn.)** | 1.008 (1.003–1.01) | < 0.001 |  |  |

LVEDP: left ventricular end-diastolic pressure; LVEF: left ventricular ejection fraction; PCWP: pulmonary capillary wedge pressure; TPG: transpulmonary gradient; Ea: PA elastance; RVEDV: right ventricular end-diastolic volume; PVR: pulmonary resistance
