# Supplementary material for: Interaction of secondary ventricular tricuspid regurgitation with RV in HFREF: an invasive pressure-volume loop study
Source: ESC Heart Fail. 2026 May 11;13(3):xvag134. doi: 10.1093/eschf/xvag134 (PMC13220961; doi:10.1093/eschf/xvag134)
Supplement: xvag134_Supplementary_Data [file xvag134_supplementary_data.zip › 54-Sensitivity Table S4 Group2.docx]

**Sensitivity analysis Group 2 (n = 23): Table S4: A higher PCWP is independently associated with a pronounced RV-PA uncoupling (Ees/Ea < 0.6) in a multivariate binary logistic regression analysis.**

|  | **Univariate** | | **Multivariate** | |
| --- | --- | --- | --- | --- |
|  | **Odds Ratio (95 % CI)** | **p** | **Odds Ratio (95 % CI)** | **p** |
| **LVEDP (mmHg)** | 1.08 (0.9–1.3) | 0.3 |  |  |
| **PCWP (mmHg)** | 1.3 (1.05–1.7) | 0.02 | 1.3 (1.008–1.77) | 0.044 |
| **TPG (mmHg)** | 1.1 (0.9–1.5) | 0.3 |  |  |
| **LVEF (%)** | 0.8 (0.7–1.03) | 0.097 |  |  |
| **RVEDV (ml)** | 1.1 (0.9–1.26) | 0.56 |  |  |
| **Age (years)** | 1.17 (1.01–1.4) | 0.031 |  |  |
| **PA compliance (ml/mmHg)** | 0.25 (0.07–0.9) | 0.038 |  |  |
| **PVR (dyn.)** | 1.007 (0.99–1.01) | 0.156 |  |  |

LVEDP: left ventricular end-diastolic pressure; LVEF: left ventricular ejection fraction; PCWP: pulmonary capillary wedge pressure; TPG: transpulmonary gradient; Ea: PA elastance; RVEDV: right ventricular end-diastolic volume; PVR: pulmonary resistance
